# Long COVID: a review and proposed visualization of the complexity of long COVID

**DOI:** 10.3389/fimmu.2023.1117464

**Published:** 2023-04-20

**Authors:** Rubeshan Perumal, Letitia Shunmugam, Kogieleum Naidoo, Salim S. Abdool Karim, Dave Wilkins, Alfredo Garzino-Demo, Christian Brechot, Sairam Parthasarathy, Anders Vahlne, Janko Ž. Nikolich

**Affiliations:** ^1^ Centre for the AIDS Programme of Research in South Africa (CAPRISA), South African Medical Research Council (SAMRC) - CAPRISA HIV-TB Pathogenesis and Treatment Research Unit, Durban, South Africa; ^2^ Department of Pulmonology and Critical Care, Division of Internal Medicine, School Clinical Medicine, Nelson R Mandela School of Medicine, University of KwaZulu-Natal, Durban, South Africa; ^3^ Long COVID Taskforce, The Global Virus Network, Baltimore, MD, United States; ^4^ Institute of Human Virology, University of Maryland School of Medicine, Baltimore, MD, United States; ^5^ Department of Molecular Medicine, University of Padova, Padova, Italy; ^6^ Division of Pulmonary, Allergy, Critical Care and Sleep Medicine and University of Arizona College of Medicine-Tucson, Tucson, AZ, United States; ^7^ Division of Clinical Microbiology, Karolinska Institutet, Stockholm, Sweden; ^8^ Department of Immunobiology and the University of Arizona Center on Aging, University of Arizona College of Medicine-Tucson, Tucson, AZ, United States; ^9^ The Aegis Consortium for Pandemic-Free Future, University of Arizona Health Sciences, Tucson, AZ, United States

**Keywords:** long COVID, long-haul COVID, post-acute sequelae of SARS-CoV-2, COVID-19, SARS-CoV-2

## Abstract

Post-Acute Sequelae of Severe Acute Respiratory Syndrome Coronavirus – 2 (SARS-CoV-2) infection, or Long COVID, is a prevailing second pandemic with nearly 100 million affected individuals globally and counting. We propose a visual description of the complexity of Long COVID and its pathogenesis that can be used by researchers, clinicians, and public health officials to guide the global effort toward an improved understanding of Long COVID and the eventual mechanism-based provision of care to afflicted patients. The proposed visualization or framework for Long COVID should be an evidence-based, dynamic, modular, and systems-level approach to the condition. Furthermore, with further research such a framework could establish the strength of the relationships between pre-existing conditions (or risk factors), biological mechanisms, and resulting clinical phenotypes and outcomes of Long COVID. Notwithstanding the significant contribution that disparities in access to care and social determinants of health have on outcomes and disease course of long COVID, our model focuses primarily on biological mechanisms. Accordingly, the proposed visualization sets out to guide scientific, clinical, and public health efforts to better understand and abrogate the health burden imposed by long COVID.

## Introduction

Severe acute respiratory syndrome coronavirus 2 (SARS-CoV-2) is a single-stranded positive-sense RNA virus that infects a range of host tissues, causing symptomatic or asymptomatic Coronavirus Disease 2019 (COVID-19) (1). As a highly transmissible airborne disease, COVID-19 poses a considerable challenge to the global public healthcare system (2). By the last quarter of 2022, there have been over 600 million confirmed COVID-19 cases and ~6.5 million deaths, corresponding to a >98% survival rate. However, for many COVID-19 survivors, recovery from the acute infection is either incomplete or temporary. Persistence of initial symptoms, recurrence of previously resolved symptoms, or the emergence of new symptoms has characterized the post-COVID journey of millions of people worldwide (3). A diversity of clinical phenotypes has been recognized, ranging from symptom clusters, discrete clinical events, organ-specific presentations, and multi-system syndromes (4–9). Epidemiological and biological risk factors for the development of Long COVID are being studied through well-characterized clinical cohorts, case-control studies, and increasingly through the application of artificial intelligence and machine learning on population-level datasets and electronic medical records (10, 11). Equally importantly, putative biological mechanisms are being sought through rigorous scientific efforts and emerging interventional trials to explain the evolution from acute infection to long-term clinical sequelae.

The condition, which has since become known as Post-Acute Sequelae of COVID-19 (PASC)/Post-COVID Condition (PCC), or Long COVID (LC, used further in this text), has emerged as a minacious new layer of the COVID-19 pandemic (12). We estimate that there are already ~100 million people suffering from Long COVID globally, for whom our limited understanding of the condition hampers diagnosis, prognostication, therapeutic intervention, and care services. Moreover, the economic burden of LC is projected to be staggering. In the USA alone, up to 4 million people have been unable to work. The total burden has been estimated at between 2.6 and 3.7 trillion dollars, including reduced health-related quality of life and earning potential (13). Over and above the disease-related disability and suffering, LC patients have faced denial and stigmatization, as well as the inability of health providers to provide coherent and efficacious treatments.

There is an evident gap in our understanding of the underlying mechanisms driving post-acute infection syndromes (PAIS). Given that PAIS may complicate recovery from almost any infectious pathogen, including parasites, viruses and bacteria, the dearth of strong scientific enquiry represents a major oversight in medical research. This has been due, in part, to the paroxysmal nature of epidemics, a high level of scientific skepticism around the validity of PAIS, historical and ongoing under-investment in this area of scientific research, and the lack of highly coordinated broadscale advocacy. High-confidence determination of the prevalence, biological drivers, clinical presentations, biomarkers, and prognosis of PAIS has been difficult due to the limited availability of data from large, prospective, well-designed studies. To date, only a few longitudinal studies have constructed population-based cohorts, raising concerns overs potential biases and generalizability. Additionally, variations caused by the implementation of different study methodologies and criteria used to establish the diagnosis of PAIS obscure comparisons of clinical status and prevalence estimates across studies. There are several viral pathogens associated with PAIS, including Ebola, Dengue, West Nile, Chikungunya, Epstein-Barr Virus (EBV), Polio, H1N1/09 (swine flu), Coxsackie B, Ross River virus, Severe acute respiratory syndrome (SARS) and Varicella-Zoster Virus (VZV). Common symptoms manifesting in as part of these post-viral syndromes include fatigue, neurocognitive/neurological deficits, sleep disturbances, myalgia, irritability, exercise intolerance, arthralgia and fever (14).

To our knowledge, there is no available framework that represents the public health importance of LC. Moreover, it is also important to consider the complex multi-nodal pathways that link acute SARS-CoV-2 infection, ***risk factors***, ***putative biological mechanisms***, and the diverse ***clinical phenotypes*** of Long COVID. This perspective review aims to provide a visualization that can be used by researchers, clinicians, and public health officials to guide the global effort toward an improved understanding of Long COVID and the provision of care to patients with the condition. We recognize that access to care, social determinants of health, and disparities may significantly contribute to the outcomes and disease course of long COVID but have narrowed our model to focus primarily on biological mechanisms considering the tremendous global variation in health disparities and because such variations should be addressed by future interventions.

## Risk factors associated with LC

The leading risk factors for developing LC are asthma (2, 15), type 2 diabetes mellitus (16), obesity (17, 18), pre-existing clinical depression, hypothyroidism (19), severe COVID-19 that required hospitalization and a greater number of symptoms during the acute infection (20). Other risk factors for LC include the presence of certain symptoms during the acute illness - shortness of breath (dyspnea) (15), fatigue (15), headaches (15, 21), anosmia (22), myalgia (15, 19) - older age (17, 19), female sex (2), socioeconomic deprivation and belonging to an ethnic minority (23). Several other contributing factors may increase the risk of developing LC, such as COVID-induced Epstein-Barr viral reactivation (24), the presence of pre-existing autoantibodies at the time of infection, and a high SARS-CoV-2 viral load during the acute infection (10).

### Severity of acute COVID-19

The onset of LC has been mainly observed in COVID-19 survivors that experienced mild to severe infection. Although it is clear that individuals who experienced asymptomatic acute infection are not protected from developing LC (2, 25), the prevalence of LC in those with severe COVID has been estimated at around 70% (26–30). Overall, while there is a gradient of risk related to the severity of the acute infection, where patients with severe acute COVID-19 are at the greatest risk for developing LC, the sheer volume of asymptomatic, mild, and moderate cases of acute COVID-19 translates to their predominance in unselected LC cohorts (29, 31–36). Therefore, the most significant burden of LC may be expected to occur among these patients. Post‐intensive care syndrome (PICS) describes a collection of health disorders that are common among survivors of critical illness, encompassing new or worsening impairments in mental, cognitive, or physical health status occurring after critical illness and persisting beyond acute care hospitalization (36, 37). Eventually, this condition will impact the long‐term prognosis, affecting both the functional outcomes and survival of patients (37). As observed in the recent pandemic, survivors of COVID-19 seem to be at a particularly high risk of developing PICS (38).

### Sex

A consistent feature of LC globally is its high prevalence in women, especially those aged 40-50 years, who are now considered highly susceptible to developing LC (26, 27). Collective data extrapolated from approximately 4,182 self-reported incident cases of COVID-19 revealed that 15% of women experienced symptoms persisting for 28 days or more, compared to 9.5% of men. In the group aged 40 to 50, women had double the risk of developing LC compared to men. A sex difference was not evident in those over 70 years (15). The immune landscape of COVID-19 patients may differ vastly between the sexes, which may underpin susceptibility to LC onset in at least some, perhaps a sizeable, fraction of women. This is because one of the leading hypotheses on LC pathogenesis posits that LC (or some of its phenotypes) is caused by immune dysregulation in the form of autoimmune disorders, which are known to be more prevalent in women than in men. Generally, more robust and rapid immune responses are seen in women, protecting them from initial infection and disease severity, including acute COVID. Contrastingly, men have a less robust immune system that dampens with age due to weakened T-cell responses (39); hence they are more likely to experience severe COVID-19 disease (30, 40). However, a robust immune response may not be beneficial in terms of LC risk and, in the long term, protective mechanisms may become overstimulated leading to immune dysregulation, ultimately resulting in the physical manifestations of LC symptoms in women (30). The molecular basis for some of this tendency towards stronger immunity and autoimmunity is now emerging. Specifically, TLR7, one of the key innate sensors of viral infections (including detection of SARS-CoV-2) is encoded on the X chromosome and is incompletely silenced in women, who then have more than one functional copy of this gene (41). Moreover, in women, increased oestrogen has been linked to decreased COVID-19 severity and mortality, presumably mediated by enhanced innate and humoral responses (42). Both of these mechanisms could contribute to autoimmune processes in LC. A study conducted in Jordan involving 495 non-hospitalised study participants between the ages of 14 and 70 revealed that female participants reported more LC symptoms than male participants. These included fatigue (61 vs 47%), anxiety (51 vs 40%), palpitations (40 vs 30%), weakness (36 vs 24%), breathlessness (33 vs 17%), and change in smell sensation (50 vs 30%) (43). In another meta-analysis of 20 studies by Maglietta et al. (2022), involving 13 340 adult patients that had been discharged after being hospitalised for COVID-19, statistically significant associations were found between female sex and any symptoms (OR 1.52, 95% [CI]: 1.27-1.82), mental health symptoms (OR 1.67, 95% [CI]: 1.21-2.29), and fatigue (OR 1.54, 95% [CI]: 1.32-1.79) (44). In a UK-based longitudinal study, meta-analysed associations from 10 cohorts encompassing 6907 LC patients revealed that women had a higher risk of LC outcomes than men (4+ weeks: OR 1.49; 95% [CI]: 1.24–1.79; 12+ weeks: OR 1.60; 95% [CI]: 1.23–2.07) (45).

### Age

Early reports suggested that older age correlated with an increased risk of LC. In a study by Sudre et al. (2021), data revealed that nearly 21.9% of patients over 70 years reported symptoms persisting for four weeks or more, compared to 9.9% aged 18 to 49 years (15). Similarly, in a multicenter prospective cohort study across Israel, Spain, Switzerland, and Italy, 20% of the patients attending COVID-19 recovery clinics were older than 65, and the older individuals were more likely to be symptomatic and more likely to experience cough and arthralgia (46). However, older age was not always found to be a risk factor for fatigue or dyspnoea, the most common LC symptoms. In contrast, a recent study determined that those aged between 30 to 39 years had a 6% lower risk, and those aged ≥70 years had a 25% lower risk compared to those aged 18–30 years, respectively (23). A recent meta-analysis, including 30 371 patients, found no significant association between age and the risk of Long COVID (OR 0.86, 95% CI 0.73 – 1.03) (47). The relationship between age and LC risk requires further investigation to characterize the complex association between advancing age, including aging of the immune system, specific biological mechanisms, and individual clinical phenotypes. Concerningly, LC may also target children, including those with asymptomatic acute infection, often resulting in extreme tiredness, shortness of breath, muscle pain, chest discomfort, palpitations, headaches and reduced cognitive abilities that often result in symptoms such as extreme tiredness, shortness of breath, last for beyond six months (30, 34, 35).

### Ethnicity

There is growing evidence that the COVID-19 pandemic has disproportionally impacted ethnic minorities and migrants in terms of cases, hospitalizations, deaths and mental health outcomes in many parts of the world. In a US-based study evaluating patients two months after COVID-19 diagnosis, Black outpatients were found to be at higher risk of experiencing LC symptoms, specifically shortness of breath, myalgia, or arthralgia, compared to ethnic minorities and Caucasian individuals (48). Heightened risks were also reported in Black Afro-Caribbean ethnic groups, mixed ethnicity and other minority ethnic groups containing patients of Polynesian, Middle Eastern or Native American origin compared to Caucasian groups (23). Similarly, in a UK-based study which evaluated patients 4-8 weeks after hospitalization for a severe COVID-19 infection, 42% of Black, Asian, and minority ethnic patients experienced shortness of breath compared to 25% of Caucasian patients. This trend was also observed in Post-Traumatic Stress Disorder (PTSD)-related LC complaints (47). A rapidly increasing concern is that vulnerable communities with partial or complete lack of quality healthcare access face challenges in convincing healthcare providers of their LC-related symptoms. Several LC-related epidemiological studies have been conducted, yet the black minority in non-African cohorts has been underrepresented, especially concerning registries, treatment regimen programs and clinical trials (23). Therefore, one critical and urgent need remains to disentangle the role of ethnicity (and genetics, see below) from the role of poor access to care in these highly vulnerable populations.

### Genetic predisposition

A remarkable finding by Zeberg and Pääbo was that a Neanderthal gene variant/haplotype on chromosome 3 significantly elevated the risk for severe COVID-19 symptoms. Possessing this specific variant conferred a 60% increased likelihood of being hospitalized (49). This variant can be found in 50% of South Asian and 16% of Europe inhabitants but is rare in African populations. In a subsequent study, utilizing data from the Genetics of Mortality in Critical Care consortium, Zeberg and Pääbo identified another Neanderthal haplotype in a region of chromosome 12 encoding oligoadenylate synthetases (OAS1-3), which, in contrast, appeared to protect against severe COVID-19 disease. Each copy of this haplotype lessened the need for intensive care-related hospitalization by 22% (50). Genes in this region encode enzymes essential to the body’s immune response to RNA viruses (51). These enzymes facilitate the breakdown of viral genomes. The genetic variability of human leukocyte antigen (HLA) molecules, which bind fragments of pathogenic microorganisms and present them to T lymphocytes to initiate or guide immune responses, can affect the susceptibility to and severity of SARS-CoV-2 infection. Severely ill patients were found to have decreased expression of HLA-DR molecules, which was associated with an immunosuppressed host response (52). Class I HLA molecules with a stronger predicted capacity to bind SARS-CoV-2 antigenic peptides were found in patients experiencing mild infection and exhibited an increased degree of heterozygosity compared to patients with moderate to severe COVID-19, consistent in their role in directing antiviral responses by CD8 T lymphocytes (53). It remains unclear if these factors are associated with an increased risk of Long COVID, independently of their association with severe disease and autoimmunity, where class II HLA molecules usually associate with susceptibility. Longitudinal studies incorporating host genetics will allow for the determination of genetic risk factors for LC.

### Immunodeficiency and immune dysregulation

A dampened or otherwise dysfunctional immunity is a crucial contributing risk factor for LC. This is often observed in individuals with malignancies or those undergoing chemotherapy, post-transplant treatment, and in conditions such as HIV/AIDS or other disorders that diminish the host immune system’s defence capabilities. Immunocompromised young adults, as well as children, are prone to prolonged viral infection, continued viral shedding, and subsequent accrual of viral variants, supporting the suspicion that weakened or inadequate host immune responses increase the possibility of SARS-CoV-2 variants emerging as a mechanism of escape to avoid immune detection, ultimately establishing conditions that favour LC onset (54–56). The prevalence of LC in PLWHA was estimated to be 43.6% (95% [CI], 33.4–54.2). Moderate to severe COVID‐19 disease was significantly associated with the post COVID condition (adjusted odds ratio, 4.7; 95% CI, 1.4–17.9; p = .016). Among PLWHA with LC, the most reported persistent symptoms were fatigue (19.1%) and cough (22.3%) (57). Elevated levels of persistent inflammation may be the underlying cause leading to the onset of LC in PLWHA. Generally, HIV is a chronic inflammatory disease associated with abnormal prolonged immune activation. Disturbances to the immune environment such as an encounter with COVID-19 may lead to a higher prevalence of LC among PLWHA. Other contributing factors that could predispose PLWHA to LC include dysfunction of the microvascular circulatory system (58), reactivation of human herpesviruses (10), and autoimmunity (59).

Another risk factor that may increase the probability of patients developing LC is the presence of lower-than-normal concentrations of specific immunoglobulin (Ig) neutralizing antibodies IgM and IgG3, in combination with other clinical risk factors such as the severity of initial acute COVID-19 infection, age and a history of asthma (60). IgM and IgG3 are the first antibodies produced in response to a new infection and are associated with boosted protection against viruses, bacteria and parasites. These antibodies were found more commonly amongst LC individuals than COVID-19 survivors without LC (10, 60, 61). Almost two-thirds of patients who develop LC have evidence of established autoantibody production at the time of COVID-19 diagnosis. The titer of autoantibodies negatively correlates with the titer of protective SARS CoV-2 antibodies such as IgM and IgG3. In combination with other clinical risk factors, an antibody-based signature may assist in determining a patient’s level of risk for LC (10, 60).

### Comorbidities

Asthma, Type 2 diabetes mellitus, hypertension, and psychological conditions such as anxiety and depression have been associated with an increased risk of developing LC. The persistence of symptoms following acute COVID-19 infection is frequently also observed in people with asthma. In a study by Buttery and colleagues (2022), 67.8% of COVID-19 asthmatic LC patients reported more frequent use of their inhaler therapy, 73.7% reported increased difficulty breathing and 59.6% reported decreased asthma control after initial COVID-19 infection. A significant proportion of patients experienced protracted asthmatic exacerbations, worsening of asthma symptoms, and the occurrence of additional symptoms besides their usual symptom profile following acute COVID-19 (62). Whether this can be explained simply by a hyperreactive airway, as is likely the case with other acute infections in asthmatics, remains to be seen.

In a study by Aminian and colleagues (2022) observed patients from day 30 to 8 months after testing positive for SARS-CoV-2, hospital admission rates were 28% higher in individuals with class II obesity (35-39 kg/m^2^) and 30% higher in class III obesity (≥40 kg/m^2^) when compared to a normal BMI range (18.5 to 24.9 kg/m^2^). This study proposed that people with moderate to severe obesity are at higher risk of LC, with an increased risk of requiring hospitalization (63). Elevated BMI is associated with greater risk of developing LC, patients are 1.031 times more likely to suffer from post-COVID-19 symptoms for each 1 kg/m^2^ increase in BMI (RR 1.031, 95% [CI]: 1.016-1.047 (64). Poorly controlled type 2 diabetes increases the likelihood of severe COVID-19 infection and is associated with an increased risk of morbidity and mortality. The COVID-19 pandemic has led to a rise in poorly controlled diabetes, progression from prediabetes to diabetes, an increase in the number of new-onset diabetes, and an escalation of corticosteroid-induced diabetes. Prolonged states of uncontrolled hyperglycemia may result in organ damage, especially to the microvascular system, which can be worsened in SARS-CoV-2 infected patients (see coagulopathy below). Post-COVID pulmonary fibrosis is more common in patients with uncontrolled diabetes. Low-grade inflammation associated with Type 2 diabetes may be aggravated during acute SARS-CoV-2 infection, leading to heightened inflammation during the post-COVID-19 period resulting in the onset of several symptoms associated with LC (16).

Pre-COVID-19 psychological distress was associated with a 1.3 to 1.5-fold increase in risk for reporting LC associated symptoms. An analysis of individuals reporting two or more LC symptoms revealed that psychological distress preceding the acute COVID-19 infection increases the risk for LC by 50%. Common symptoms reported in such individuals included fatigue, digestive, neurological, cardiovascular, respiratory, and cognitive symptoms present for more than four weeks after acute infection. Psychological distress was linked to a 15-51% greater risk of daily life impairment due to LC consequences, with 56% of individuals reporting at least occasional daily life impairment relating to the condition (65).

Early analysis from the SARS-CoV-2 pandemic revealed that hypertension is the most shared pre-existing clinical condition observed amongst hospitalized COVID-19 patients, affecting 30-50% of global cohorts. Hypertension has now also been implicated in the onset of LC. A case-control study conducted by Fernández-de-las-Peñas (2022), a first of its kind to investigate the link between hypertension and LC, revealed that hypertensive participants demonstrated a substantially larger number of LC symptoms (66). The mechanism underlying LC development in hypertensive individuals is still not fully understood.

## Biological mechanisms of LC

### Viral persistence and persistent immune activation

Since the start of the pandemic in 2020, there has been significant effort to provide information on potential biological mechanisms influencing the viral activity and overall pathogenicity of SARS-CoV-2. Persistence of the virus or its components is a highly plausible biological mechanism for LC. Studies have demonstrated the persistence of viral antigens, viral RNA and whole virus in the brain, sinus, adrenal glands, kidneys, gut, lymph nodes, spleen, lungs and heart (67). Viral persistence may result in symptoms through (i) direct viral cytopathic effects; (ii) regional inflammation; (iii) provoking a.n immune response causing an elevated and prolonged state of inflammation; and (iv) prompting autoimmunity (54, 68).

Viral persistence may be associated with a high degree of immune activation during acute SARS-CoV-2 infection (69). COVID-19 severity is influenced by dysfunction and dysregulation of the host immune response resulting from disturbance of the type I interferon pathway in acutely SARS-CoV-2 infected host cells. Reduced or delayed host immune signaling responses would be expected to favor viral persistence and the potential production of SARS-CoV-2 variants (54). Furthermore, SARS-CoV-2 has been shown to contain superantigen-like molecules that may overstimulate T cells, resulting in a cytokine storm, but eventually also in T cell exhaustion and a weakened capacity for viral clearance, inadvertently allowing SARS-CoV-2 to persist (70). Additionally, viral mimicry of self-proteins may enable it to evade immune surveillance and viral clearance. It should also be highlighted that LC exhibits autoimmune disease characteristics (see autoimmunity below); SARS-CoV-2 infected individuals may be more prone to the onset of LC owing to further activation of an already aggravated memory immune response that cannot be sufficiently modulated by mechanisms of homeostasis when the viral infection is finally cleared (71).

### Autoimmunity

Autoimmunity is emerging as an increasingly plausible biological mechanism for at least some LC phenotypes. The prevalence of autoantibodies in LC patients is elevated compared to the general population (72). Severe COVID has shown striking similarity to systemic autoimmunity seen in systemic lupus erythematosus (SLE), with marked extrafollicular B cell maturation and autoimmune autoantibody production by double-negative (CD27- IgD-) B cells (73). The resultant autoantibodies target specific extractable nuclear antigens such as LA/SS-B, Ro/SS-A, Jo1, P1 and U1-SnRNP, further reminiscent of SLE (72, 74). Auto-antibodies to interferon type 1, in up to 10% of severe COVID cases, would also be expected to limit the host immune system’s ability to effect viral clearance appropriately, but their role in LC remains speculative at this time. More recently, multi-dimensional immune profiling of 99 LC patients by Rapid Extracellular Antigen Profiling, a high throughput method capable of measuring antibody reactivity to >6000 extracellular and secreted human proteins, failed to identify any stereotypical autoantibodies to distinguish LC patients from healthy controls (75). However, differential antigen targeting in the LC and control groups revealed shared patterns of autoreactivity in a subset of LC patients with tinnitus and nausea. Moreover, it remains unclear whether antibodies to intracellular autoantigens and non-protein autoantigens are implicated in the pathogenesis of Long COVID.

### Microclots

There is rapidly growing evidence to support the presence and persistence of microclots in individuals with LC relative to those without prior COVID-19 infection (58). A similar phenomenon has previously been demonstrated in individuals with type 2 diabetes (76). These LC-associated microclots are (i) resistant to fibrinolysis; (ii) harbour antitrypsin rendering them resistant to lysis processes; (iii) encompass inflammatory molecules, which may prompt inflammation upon lysis; and (iv) postulated to result in the occlusion of the microcirculation in various capillary beds triggering tissue dysfunction and ultimately resulting in organ dysregulation and dysfunction, culminating in clinical presentations related to an organ system (77, 78).

### Persistent CNS dysfunction

In COVID-19 patients, it was proposed that the SARS-CoV-2 virus enters the host’s upper respiratory tract causing damage to the olfactory mucosal cells and the loss of smell and taste. The virus would then travel toward the brain through axonal transport or *via* the lymphatic system. This event would be expected to induce an inflammatory glial reaction (79) that, with or without a CNS-targeted autoimmune response, could contribute to the overall CNS effects with symptoms such as extreme tiredness, headaches and cognitive impairments, including memory loss, lack of concentration and brain fog (80, 81). Tachycardia, palpitations and chest discomfort are common symptoms of LC in approximately 20 to 40% of COVID-19 survivors. The brain’s respiratory and cardiovascular control centres are closely intertwined in the brainstem, in regions such as the caudal ventrolateral medulla (CVLM), ventral respiratory column (VRC) and the pontine respiratory group (PRG). The CVLM is made up of neurons responsible for regulating heart rhythms. The VRC controls rhythmic breathing with coordinated cycling between inspiration and expiration. The PRG modulates the transition between expiration and inspiration. Thus, disturbances in this region can steadily result in dysautonomic phenotypes. SARS-CoV-2 may also enter the brainstem directly *via* the surface expression of ACE-2 (82) because the brainstem has a relatively high expression of ACE-2 receptors compared to other brain regions (79, 83). These events would lead to the generation of inflammatory mediators that damage vascular cells and the brainstem. Microthrombosis resulting from vascular injury may further potentiate neuroinflammation and brainstem dysfunction. Thus, continuous brainstem dysfunction at the level of CVLM, VRC and PRG may explain LC’s prominent cognitive and cardiorespiratory-related symptoms (82).

### Persistent vascular inflammation

Thromboinflammation is mainly triggered by the interplay between SARS-CoV-2 and the humoral innate immune system, specifically by an interplay between coagulation, fibrinolytic and complement systems. Activation of and/or damage to endothelial cells, leukocytes and platelets all funnel into thrombotic and inflammatory reactions (84). During LC, the ongoing endothelial dysfunction combined with an impaired innate immune response may promote the onset of an acute inflammatory reaction of the endothelium, potentially leading to acute myocardial injury, myocarditis, and thromboembolic complications affecting both the venous and arterial circulation (36). SARS-CoV-2 mediated endothelial dysfunction may also induce excessive production of thrombin, fibrinolysis inhibition, and continuous activation of the complement pathways resulting in prolonged dysfunction of the microvascular system and microthrombosis (85). Microvascular thrombosis occurs systemically and can affect several organs, especially those with high capillary density, such as the lungs (86). The mechanisms underlying the association between LC and the onset of cardiovascular diseases are not yet fully understood. However, several mechanisms have been proposed, including continuous damage directly induced by viral invasion and subsequent death of cardiomyocytes, infection of endothelial cells, endothelialitis (87), transcriptional alteration of cardiomyocytes, complement activation and complement-mediated coagulopathy and microangiopathy (88), ACE-2 downregulation, renin–angiotensin–aldosterone system dysregulation (89), autonomic dysfunction (90), increased concentrations of pro-inflammatory cytokines (91) and transforming growth factor β (TGF-β)/SMAD signalling pathway activation inducing fibrosis of cardiac tissue (92).

### Residual tissue damage

Persistent symptoms in LC patients may arise because of tissue or organ injury induced by abnormal clotting or inflammatory during acute COVID-19 infection. In a Swiss-based prospective observational cohort study involving adult patients that survived acute COVID-19 following mild/moderate or severe/critical COVID-19, substantial functional and radiological abnormalities suggestive of lung parenchymal and small airway disease was observed 4 months after the initial infection (93). It has also been noted that more than one third of recovered COVID-19 patients develop lung abnormalities associated with fibrosis, following hospitalisation (94). Risk factors associated with the development of post-COVID-19 lung fibrosis include advancing age, prolonged ICU stay and duration of mechanical ventilation, excessive alcohol consumption, smoking, severe COVID-19, and a high burden of comorbidities including coronary artery disease, diabetes and hypertension (93–96). Mechanical ventilator-induced lung injury (VILI) poses an additional risk due to the abnormal alveolar pressure or volume dynamics that cause lung tissue injury leading to the generation of proinflammatory modulators thus exacerbating acute lung injury, facilitating increased pulmonary fibrosis in those that survive. Patients with a history of cigarette smoking exhibited a much higher incidence of post-COVID-19 lung fibrosis compared to non-smokers. In a cross-sectional prospective study conducted in Egypt, out of 30 smoking patients, 18 developed post-COVID-19 pulmonary fibrosis (60%) (95). Post-COVID lung fibrosis has been documented as a probable perturbing sequela among COVID-19 survivors, in which permanent structural distortion within the lung tissue environment may occur leading to irreversible dysfunction (95).

Biological mechanisms underlying cardiovascular injury in the onset of LC are still not yet full elucidated. Postulated mechanisms include persistent damage as a result of direct invasion of cardiomyocytes by SARS-CoV-2 followed by cell death, viral infection of endothelial cells and endotheliitis, transcriptional alteration of multiple cardiac cell types, complement activation and complement-mediated coagulopathy and microangiopathy, ACE2 downregulation and deregulation of the renin–angiotensin–aldosterone system, dysautonomia, increased pro-inflammatory cytokines production and release and activation of Transforming growth factor β (TGF-β) signaling *via* the Smad signalling pathway (91). TGF-β stimulates fibroblasts and accelerates extracellular matrix production within injured tissues. Disproportionately excessive aggregation of extracellular matrix, results in fibrosis and eventually scarring of cardiac tissue (91, 96). Another proposition is that a chronic inflammatory response is induced by prolonged viral reservoirs in the cardiomyocytes following acute infection. This condition may be further aggravated by obesity, which stimulates activation of the inflammatory signalling pathway mediated by c-Jun N-terminal Kinase (JNK) and nuclear factor-kappa B (NF-κB) (97). Downstream effects lead to the generation of multiple pro-inflammatory cytokines in adipocytes such as monocyte chemoattractant protein-1 and Regulated upon Activation, Normal T Cell Expressed and Presumably Secreted (RANTES), chemokines that exacerbate endothelial dysfunction *via* endothelial nitric oxide synthetase uncoupling and production of reactive oxygen species, which promotes insulin resistance and pro-inflammatory macrophages infiltration (97, 98). Subsequently, these processes culminate in the unsolicited detrimental damage of cardiac tissue, advancing to chronic fibrosis of the myocardium and compromised ventricular compliance, diminished myocardial perfusion, intensified myocardial stiffening, leading to decreased cardiac contractility and an increased likelihood of arrhythmias (98).

### Reactivation of latent viruses

SARS-CoV-2 infection-induced reactivation of latent herpesviruses as a possible contributing factor to LC has been studied. In a study of 215 individuals with Long COVID, unexpected increases were observed in antibody responses directed Epstein-Barr virus (75). Also, in a cohort of 280 adults with prior SARS-CoV-2 infection, Peluso et al. observed that LC symptoms, such as fatigue and neurocognitive dysfunction, at a median of 4 months following initial diagnosis, were associated with serological evidence suggesting recent EBV reactivation (early antigen-diffuse IgG positivity) or high nuclear antigen (EBNA) IgG levels but not with ongoing EBV viremia. Serological evidence suggesting recent EBV reactivation (early antigen-diffuse IgG positivity) was most strongly associated with fatigue (OR = 2.12). In this study, underlying HIV infection was also associated with neurocognitive LC (OR = 2.5). However, those with serologic evidence of prior CMV infection were less likely to develop neurocognitive LC (OR = 0.52) (99). In another study the authors tested for SARS-CoV-2 RNA in stool and throat washings, and EBV DNA in stool, throat washings and blood by real-time PCR (EBV) and real-time RT-PCR (SARS-CoV-2) in 30 LC patients characterized by persistent fatigue, post-exertional malaise (PEM), autonomic dysfunction and/or orthostatic intolerance and who all had had a mild SARS-CoV-2 infection. Twenty age- and sex-matched patients who had fully recovered after the SARS-CoV-2 infection served as controls. SARS-CoV-2 RNA indicating persistent infection was not detected in throat washing nor stool. SARS-CoV-2 antibody titres (IgA and IgG) did not differ between the cohorts. EBV real-time PCR was negative in all blood or stool samples. However, EBV DNA was detected in throat washings in 15/30 (50%) of LC patients, while in only 4/20 (20%) of non-LC patients (*p* = .0411) (100). A study of patients with myalgic encephalomyelitis/chronic fatigue syndrome (ME/CFS) and healthy donors as controls analyzed whether a mild/asymptomatic SARS-CoV-2 infection imposes latent EBV, HHV6 and HERV-K virus reactivation. At 3-6 months after infection, virus-specific antibodies in saliva were substantially induced, indicating a strong reactivation of all three viruses in both cohorts. In patients with ME/CFS, however, antibody responses were significantly stronger, in particular against EBV-encoded nuclear antigen-1 (EBNA1) than in the controls (101).

The role, if any, reactivated EBV infection may have on the development of LC remains to be fully elucidated.

### Persistent metabolic dysfunction

The metabolic sequelae of SARS-CoV-2 infection are highly complex, involving the dysregulation and dysfunction of multiple organs. Metabolic dysregulation is thought to be driven by immunopathological events, including the overproduction of inflammatory molecules leading to persistent inflammation and incomplete systemic recovery. Closely related coronavirus diseases, such as Middle East Respiratory Syndrome (MERS) and SARS, have been shown to induce long-term metabolic modifications in convalescent patients resulting in liver disorders, altered glucose metabolism and hypertriglyceridemia. Several studies have demonstrated the presence of persistent metabolic derangement in SARS-CoV-2 infection, suggesting this mechanism is one of the underlying routes of LC pathogenesis (102). The earliest bioenergetic effects of SARS-CoV-2 infection occur through the binding of viral polypeptides to critical host cell mitochondrial proteins, retarding ATP production. Subsequently, SARS-CoV-2 produces a pervasive inhibition of the transcription of key mitochondrial genes central to cellular respiration (103). Mitochondrial dysfunction and resultant bioenergetic failure have been demonstrated during acute infection. The same is true of lipid metabolic dysbalance, including the production of phospholipases that were found to be predictive of poor outcomes (104). However, establishing impaired cellular respiration and/or lipid dysregulation in the context of LC requires further research (105, 106).

### Dysbiosis

Recently, evidence has emerged to propose the role of the lung‐gut axis in increasing the risk for severe COVID-19 (107). Gut microbiota dysbiosis correlates with poor clinical outcomes in mechanically ventilated COVID‐19 patients. These findings indicate a strong interrelation between SARS‐CoV‐2 consequences and host gut microbiota, which may also be associated with an increased risk of LC. The substantial rise in pathogenic bacteria and simultaneous reduction in anti‐inflammatory microbiota promotes sustained intestinal inflammation during COVID‐19, subsequently leading to prolonged recovery (108). Su and colleagues (2022) followed 155 patients for 14 months after being diagnosed with acute COVID-19 infection; 76.4% were diagnosed with LC at six months and 78.7% at 14 months. In LC-associated dysbiosis three most common symptoms were disrupted sleep (35.5%), cognitive difficulties relating to memory (44.5%) and fatigue (50.9%). It was found that the gut microbiota of the LC patients did not fully recover at one year in after COVID-19 diagnosis (109); genera *Prevotella* and *Veillonella* dominated the oral microbiome of COVID-19 patients. The *Prevotella genus* produces proteins that can promote SARS-CoV-2 infection and can increase disease severity. These genera have also been associated with an elevated risk of pneumonia-induced mortality in frail, older patients. Both genera are known inducers of inflammatory responses. *Prevotella* strains mainly activate TLR-2 and boost the expression of inflammatory cytokines IL-1 and IL-23. *Veillonella* species is a potent inducer of IL-6. Several other pro-inflammatory microbiotas may be responsible for prolonged disease symptoms, such as multiple *Actinomyces species, S. moorei* and *L. wadei*. In LC patients, the study demonstrated that metabolic pathways accompanying the generation of pro-inflammatory molecules were abundantly increased. In contrast, anti-inflammatory pathways were downregulated, which may be one of the main attributes underlying the persistence of LC and associated symptoms (110). Beyond direct disturbance of the microbiome, evidence is accumulating to suggest that mucosal barrier breach and microbial component/product translocation into the systemic circulation may occur in LC (“leaky gut”). This includes specific evidence of fungal translocation as measured by systemic beta-glucan increase, which has been detected in 74% of LC cases by a recent study (111).

### Amyloidogenesis and postural orthostatic tachycardia syndrome

Amyloidogenesis is a speculative mechanism for some LC symptoms (112), including cognitive, behavioral, and psychiatric symptoms resembling neurodegenerative disorders. Many neurodegenerative diseases, such as Parkinson’s and Alzheimer’s are distinguished by the abundance of amyloid or amyloid plaques (113). Amyloid fibrils of the SARS-CoV-2 spike protein have been localized in nerve tissues with heterologous seeding promoting the accumulation of endogenous proteins, which may lead to neurodegeneration (114). In COVID-19 patients, blood clotting is related to extracellular amyloidotic fibrillar assemblages. Hence, when plasma from healthy donors was incubated with the SARS-CoV-2 spike protein, hypercoagulation and impaired fibrinolysis were demonstrated. Previous studies have linked amyloidosis to thromboinflammation, activation of surface-activated coagulation system (FXII Kallikrein/Kinin), disruption of the fibrinolytic system, dysregulation of blood coagulation and cerebral amyloid angiopathy; collectively, suggesting associations between COVID-19 phenotypes and S-protein amyloidogenesis. A study conducted by Nystrom and Hammarstrom (2022) found amyloidal sequences in SARS-CoV-2 spike protein and that endoproteolytic cleavage of the protein resulted in amyloid formation (114). Amyloidosis, resulting from the systemic deposition of amyloid proteins, may present as localized or systemic conditions with many generalized or organ-specific phenotypes overlapping with the reported LC clinical phenotypes (114)

Some symptoms observed in LC patients suggest immune or virus-induced autonomic nervous system (ANS) disruption, resulting in temporary or possibly long-term orthostatic intolerance. Many have hypothesized that COVID-19 infection affects the ANS (see the above discussion of autonomous centers in the brain stem and accessibility to SARS-CoV-2) and that the SARS-CoV-2 virus itself may mediate the resultant autonomic dysfunction. POTS involves many symptoms such as nausea, heart palpitations, blurry vision, headaches, tiredness, cognitive impairments and lightheadedness. The exact cause underlying the development of POTS in LC remains unknown, but dysautonomia has been commonly implicated. Autonomic disorders like POTS are often associated with abnormal activity of muscarinic receptors and alpha and beta (α/β) adrenoreceptors. Usually, when a healthy individual stands, blood accumulates in the pelvis and limbs, which causes a reduction in venous return to the heart. Cardiac baroreceptors sensing the volume change, stimulate an increase in sympathetic adrenergic and neural tone modulated by epinephrine and norepinephrine, respectively. Consequently, these events lead to tachycardia and splanchnic vascular bed vasoconstriction for elevated venous cardiac return. During orthostatic intolerance, norepinephrine and epinephrine release induces distinct tachycardia, manifesting as chest pain, shortness of breath and palpitations; symptoms frequently associated with LC Extremely high catecholamine levels can cause inconsistent vasodilatation, withdrawal of sympathetic activity and vagus nerve activation, ultimately resulting in dizziness, blood pressure instability and fainting (115–117).

## Clinical characterization of LC

The World Health Organization (WHO) consensus case definition asserts that LC may occur in individuals with a history of presumed or confirmed SARS-CoV-2 infection, usually three months from the initial onset of the infection with symptoms continuing for at least two months accompanied by the inability to explain the symptoms through an alternative diagnosis (118). Many COVID-19 survivors report at least one or more virus-related symptoms that persisted for more than four weeks following the initial diagnosis (60). Long COVID was observed in 54% of hospitalized patients and 34% of non-hospitalized COVID patients (2). Furthermore, the risk of developing Long COVID increases with each subsequent reinfection with SARS-CoV-2 (119).

Common LC phenotypes are fatigue, anosmnia/dysgeusia, respiratory (shortness of breath and coughing), cardiovascular (major adverse cardiovascular events and arrhythmias), cognitive (brain fog, impaired memory and concentration), neurological (headaches, dizziness, insomnia, excessive daytime sleepiness, and postural orthostatic tachycardia syndrome), psychiatric, musculoskeletal (myalgia, costochondritis and arthralgia), coagulopathy (deep vein thrombosis, pulmonary embolism and bleeding), dermatological (COVID toes, urticaria and skin rashes), and gastrointestinal (diarrhea, abdominal pain and reflux).

### Diverse clinical phenotypes

#### Fatigue

Fatigue is the most reported LC symptom, occurring in between 11 to 87% of patients, depending on the severity of the initial infection and the time from symptom onset (75, 120). The prevalence of fatigue declines over time, yet as much as 60% of previously hospitalized patients report ongoing moderate to severe fatigue up to one year after the initial infection (121). In a study evaluating self-reported neurocognitive Long COVID, the severity of fatigue was found to be higher in non-hospitalized patients, in whom it was also more likely to be associated with other long-term sequelae (122). Two distinctive patterns of fatigue have become evident: chronic fatigue and post-exertional fatigue. While chronic fatigue is characterized as a constant extreme lack of energy, post-exertional fatigue describes a pervasive intolerance to physical, mental, emotional, or environmental stressors (123). The development of post-viral fatigue is associated with postural orthostatic tachycardia syndrome (POTS), autoimmunity, metabolic dysfunction/bioenergetic failure, reactivation of latent viruses, and viral persistence. Fatigue may occur in isolation but is more frequently reported in association with anxiety, depression, pain, cognitive impairment, cough and dyspnea (5, 124) Using hierarchical clustering to characterize LC phenotypes, fatigue was located within a cluster in which pain symptoms, such as myalgia, joint pain, and headaches, were most frequent (5).

#### Anosmia/dysgeusia

A diminished sense of smell and/or taste has been a characteristic, although not pathognomonic, feature of LC, affecting 10 to 20% of patients. The prevalence of anosmia and ageusia decreases over time, but ~3 to 5% of patients report the persistence of the symptoms up to one year after the initial infection (5, 120, 125). Patients with these symptoms do not generally experience debilitating pain, cognitive difficulties, or fatigue and demonstrate normal functional status. Anosmia and ageusia have been linked to viral persistence and persistent CNS dysfunction.

#### Respiratory

Persistent dyspnea occurs in 10 – 30% of patients following acute COVID-19 and in up to 60% of patients with LC. There is a clear gradient of risk proportional to the severity of acute illness, and the dyspnea may persist without significant improvement over time (121, 125). Shortness of breath is commonly accompanied by fatigue, cognitive difficulties, and chest pain (5). In addition to residual pulmonary damage from the acute infection, implicated biological mechanisms for persistent dyspnea include autoimmunity, pulmonary vascular microclots with diminished pulmonary perfusion, diaphragmatic weakness and nervous system dysfunction (central and autonomic) (75, 126–128).

#### Cardiovascular

Palpitations and chest pain occur in 5 -10% of previously hospitalized patients following acute infection and commonly occur alongside fatigue, dyspnea and cognitive difficulties (125, 129, 130). Unexplained resting tachycardia, or an exaggerated heart rate response to exercise, has also been described. In postural orthostatic tachycardia, which has been reported following COVID-19, there is an inappropriate increase in heart rate in response to standing (131, 132). Moreover, there is an increased long-term risk of major adverse cardiovascular events and arrhythmias following acute COVID-19 (4, 91). There is evidence of structural changes to the heart after COVID-19 infection, best demonstrated by magnetic resonance imaging, and putative mechanisms include autoimmunity, microclots in the coronary circulation, and viral persistence in the myocardium (78, 133–135). In a cohort of patients who previously experienced mild or moderate COVID-19, and who subsequently presented with angina-like persistent chest pain as part of their Long COVID sequelae, adenosine stress perfusion cardiac MRI was strongly suggestive of coronary microvascular ischemia as the cause of the cardiac symptoms (136). Cardiovascular dysfunction associated with LC may result from arterial stiffening, endothelial injury and a persistently high oxidative burden (137).

#### Coagulopathy/vascular events

A persistently increased risk for coagulopathy following acute COVID-19 may result in discrete clinical events of deep vein thrombosis (DVT), pulmonary embolism (PE) or bleeding. Coagulopathy has been linked to persistent endothelial activation, platelet dysfunction and microclots, autoimmunity (particularly antiphospholipid antibodies), the persistence of neutrophil excitation traps, and viral persistence (78, 119, 134, 135, 138, 139). As reported, there are significantly increased incidence rates and risk of DVT up to 70 days after covid-19 infection, PE up to 110 days, and bleeding events up to 60 days, coupled with the risk of PE in the acute phase being exceptionally high (139). In a study that analyzed data on vascular events (VEs) following COVID-19 diagnosis, the increased incidence of VEs following COVID-19 diminishes faster for arterial thromboses than venous thromboembolic events. However, the incidence of VEs remains a high risk for up to 49 weeks after acute infection (140).

#### Cognitive

Cognitive difficulties have been widely reported in LC cohort and may occur in 2 – 10% of patients following acute COVID-19 and up to 35% of patients with LC. The symptoms may manifest as difficulty in concentrating, memory impairment, or even more complex cognitive deficits. LC has been associated with objective declines in cognitive abilities, a reduction in general intelligence, and structural brain changes to anatomical and functional areas, which map to the most commonly described cognitive symptoms (81, 141–143). Cognitive deficits in LC have been attributed to persistent central nervous system inflammation, autoimmunity, viral persistence, and cerebral circulation microclots with diminished brain perfusion (102, 135, 141, 143–145).

#### Neurological

Neurological symptoms have been frequently observed in LC patients, including insomnia (27.4to 49.6%)%), excessive daytime sleepiness (35.8%), anxiety (19.1%), post-traumatic stress disorder (15.7%) and depression (12.9%) (146). Many global cohorts reported an exceptionally high prevalence of insomnia, and mental and emotional disorders in the first six months after COVID-19 infection (147). Patients that survive the first 30 days of the acute SARS-CoV-2 infection have a significantly increased risk of experiencing neurological episodes or developing neurological disorders. A study by Xu et al. (2022) revealed a 42% increased risk of developing a neurological sequela 12 months following acute COVID-19 infection, which translates to an overall 7% burden of infected individuals (148). This observation was most evident in subgroups with immune dysfunction, hypertension, chronic renal disease, diabetes, hyperlipidemia, poor socioeconomic status, current smoking, and obesity (148).

#### Psychiatric illnesses

There is mounting evidence associating LC with an increased risk of new-onset psychiatric illnesses, mainly attributed to its impact on the brain. LC has also been associated with elevated suicide risk. Acute psychotic disorders following recovery from acute COVID-19, disorganized behaviour, Cotard’s delusion and a potentially high risk of psychotic homicide have all been described (149). In a monocentric cross-sectional cohort study by Gasnier and colleagues (2022), 177 patients admitted into the ICU during acute infection in the first wave of the pandemic and reporting LC symptoms were assessed four months after hospitalization (150). Approximately 65% of LC patients experienced at least one psychiatric symptom within six months following acute COVID-19 diagnosis. Symptoms of LC psychosis include disrupted sleep, paranoid delusions, and hallucinations. Prominent respiratory symptoms during the acute infection, irrespective of the need for hospitalization, were associated with a higher risk of new-onset psychiatric disorders. Cognitive deficits were also associated with an increased risk of new-onset psychiatric disorders (150). Psychiatric symptoms may rapidly improve or completely resolve with appropriate early intervention and treatment using antipsychotic agents. However, this may not apply to all individual LC patients, some of whom report minimal improvement after six months of follow-up.

#### Gastrointestinal

In a cohort of COVID-19 survivors, 29% of LC patients reported gastrointestinal-related symptoms at six months, including reflux (16%), nausea or vomiting (7%), abdominal pain (9%), constipation (11%) or diarrhoea (10%) (151). It has been hypothesized that this may be partly due to the gut epithelium having a high expression of the ACE2 receptors, rendering it a site of heightened viral activity. Additionally, widespread inflammation caused by the viral infection can also disrupt the gut microbiome, thereby inappropriately stimulating the regional nerves and causing abnormal gastrointestinal behaviour (9, 152, 153).

#### Musculoskeletal

A high prevalence of persistent musculoskeletal and rheumatic symptoms has been reported among LC patients. At three months following the recovery from COVID-19, 89% of LC patients reported at least one lingering symptom, while 75% had at least one musculoskeletal or rheumatic symptom (154). At six months, 60% of previously hospitalized patients with acute COVID-19 that did not require intensive care reported at least one persistent symptom, while 43% had a minimum of one musculoskeletal or rheumatic symptom. The most common musculoskeletal and rheumatic symptoms observed in LC patients include myalgia (15.1%), joint pain (18.6%) or fatigue (31.6%). Other persistent COVID-19 symptoms reported in these patients were shortness of breath (25.3%), hair loss/alopecia (20%) and diaphoresis (17%) (154). Patients with LC may also present with costochondritis, a benign form of chest wall pain caused by costal cartilage inflammation, often accompanied by severe and difficult-to-treat coughing (155, 156). Arthralgia has been extensively described as a common symptom of LC, estimated to occur in at least 1 in 5 patients (157). Musculoskeletal LC-associated symptoms may linger up to 8 months after recovery from COVID-19, with most patients developing *de-novo* join or muscle pain (154).

#### Dermatological

Among LC patients, various dermatologic manifestations may arise due to infection with SARS-CoV-2, which may be related to the activation of inflammatory pathways. The median range of days for the following dermatological conditions are as follows: Morbilliform exanthema 4-7 days; urticarial flareups 4-28 days; papulosquamous outbreaks 20-70 days; pernio lesions 60-130 days; while pernio with associated livedo reticularis continued for more than 150 days in some LC patients (158). Chronic Acral Lesions, also known as COVID toes, most likely result from host immunity against SARS-CoV-2 and the associated production of type I interferon. This condition may persist for 3 to 10 months and is associated with symptoms ranging from no pain to digital incapacity (159).

### Development of new chronic diseases

Individuals previously hospitalized with severe COVID-19 infection are more likely to develop type 2 diabetes mellitus, chronic renal disease (CRD), chronic liver disease, coronary artery disease or organ failure due to SARS-CoV-2 activity within host tissues. Interestingly, organ impairment was also observed in non-hospitalized COVID-19 infected patients that did not require supplemented oxygen (160, 161). Individuals with Long COVID exhibited an increased risk and excess burden for diabetes in the 12 months following acute infection. Risks and burdens of post-COVID outcomes increased in a graded fashion correlating with acute disease severity (162). Patients at high risk for developing CRD included those with frailty, chronic illnesses, disability, and immune senescence (163). Liver injury in COVID-19 patients is associated with prolonged hospitalization and acute severe disease. Among critically ill adult COVID-19 patients, the incidence of hyperbilirubinemia is increased by 1.7-fold, and indirect markers of liver injury, including hypoalbuminemia, are raised by 7-fold (164). Recovered COVID-19 patients with critical cardiopulmonary involvement may show clinical and histological features of severe cholangitis and intrahepatic microangiopathy. Notably, severe LC-associated cholangiopathy increases the risk for long-term hepatic morbidity (165). Patients surviving beyond the first 30 days after infection have an increased incidence risk of cardiovascular disease across several categories, including thromboembolic, heart failure, myocarditis, pericarditis, non-ischemic and ischemic heart disease, dysrhythmias and cerebrovascular events. Overall, the risk and 1-year burden of cardiovascular disease in survivors of acute COVID-19 are substantial for those presenting with COVID-19 beyond the first 30 days of infection (137).

### The impact of vaccines on the risk of long COVID

In vaccinated patients experiencing a breakthrough episode of COVID-19, first and second dose vaccination was associated with reduced likelihoods of requiring hospitalization or developing more than five symptoms in the first week of infection. For those who had two vaccine doses, the odds of long-duration symptoms (≥28 days) was significantly reduced compared to unvaccinated individuals. In a study by Antonelli et al. (2022), most persistent symptoms following acute COVID-19 were reported less often in vaccinated individuals than in unvaccinated individuals (166). At-risk individuals remain highly susceptible to infection with SARS-CoV-2 despite vaccination due to immune-senescence, multimorbidity (diabetes, hypertension, and obesity) and poor socioeconomic status. Nonetheless, vaccination in this vulnerable population is associated with lower mortality risk (HR = 0.66, 95% CI: 0.58, 0.74) and a significantly lower risk of Long COVID (HR = 0.85, 95% CI: 0.82,0.89) (4, 167). Notably, vaccination was observed to have reduced the risk of Long COVID symptoms such as sleeping disturbances, myalgia, renal injury and cognitive deficits, in individuals vaccinated either before or after acute infection (168). The probability of persisting Long COVID symptoms decreases steadily following vaccination, with a growing body of evidence suggesting that lasting improvements are observed after a second vaccine dose (169). Azzolini et al. (2022) revealed the association between the number of BNT162b2 vaccine doses and Long COVID in healthcare workers who did not require hospitalization, providing evidence that the number of vaccine doses received was inversely related to the incidence of Long COVID: unvaccinated 41.8% (95%CI, 37.0%-46.7%), one dose vaccination 30.0% (95% CI, 6.7%-65.2%), two-dose vaccination 17.4% (95%CI, 7.8%-31.4%), and three-dose vaccination 16.0%(95%CI, 11.8%-21.0%) (167). The impact of the COVID-19 vaccination on people presenting with Long-COVID still remains incompletely understood, with conflicting reports from existing studies.

## The proposed visualization of long COVID and its utility

### Complexity

**Figure 1:**
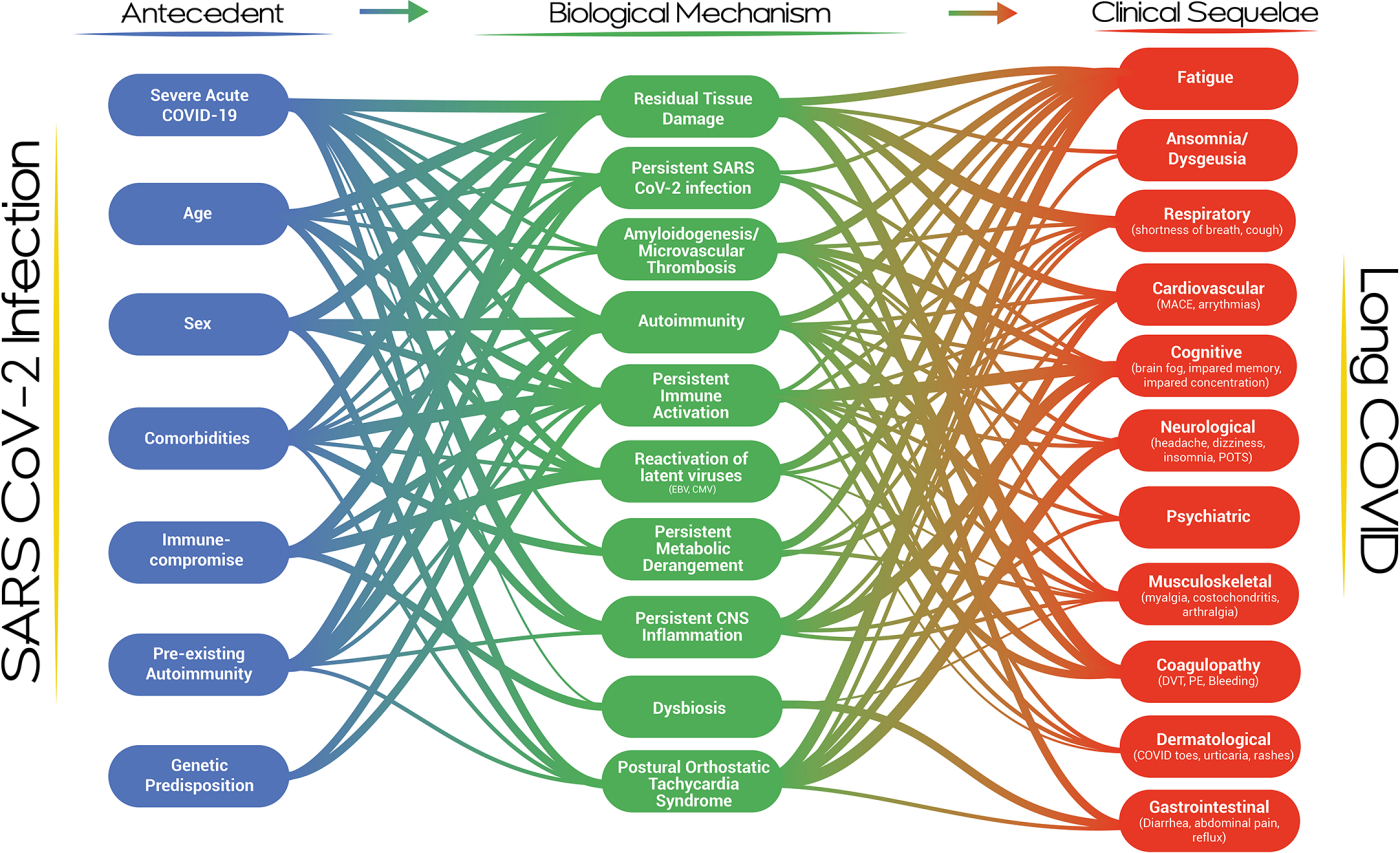
A systems-level visualization of the complexity of Long COVID and its pathogenesis.

**Figure 2:**
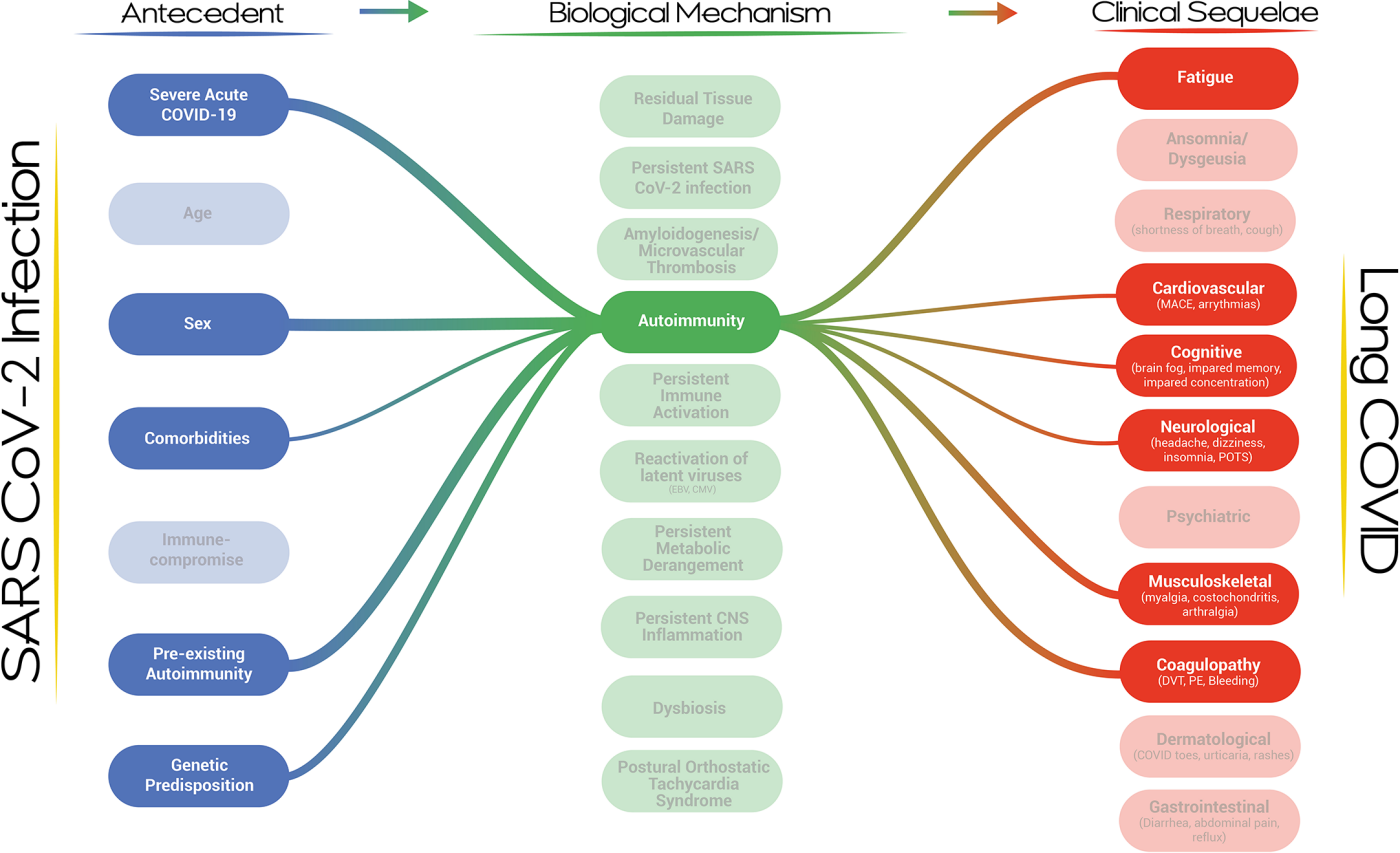
A network map of the risk factors and clinical sequelae associated with autoimmunity in the context of Long COVID.

Our proposed visualization of Long COVID and its pathogenesis presents a dynamic, modular and systems-level approach to the condition (**Figure 1**). The visualization provides an overview of the current thinking and evidence establishing the multi-nodal connection/s between acute SARS-CoV-2 infection and clinical phenotypes. It incorporates risk factors and biological mechanisms as essential nodes in the development of the Long COVID. The strength of the association between any two nodes, based on a qualitative synthesis of the current evidence by the authors, is represented by the thickness of the connecting lines. It is apparent that a single pathway is unlikely to explain the genesis and evolution of this complex post-viral phenomenon and that multi-hit, multi-mechanistic pathways are more likely. Patients may present with distinct and easily identifiable phenotypes or overlapping/multi-system phenotypes, with the evolution of the clinical phenotypes over time. The visualization is capable of expressing this complexity by demonstrating that pathways may evolve or may emerge and abate dynamically in an individual patient. The propensity of a particular pathway to alter with time is the subject of research in several Long COVID cohorts. It is also apparent that clinical phenotypes may result from a particular biological mechanism or as a result of multiple biological mechanisms acting in parallel or in series over time. It is equally apparent that a single biological mechanism may result in diverse clinical phenotypes, especially when the mechanism is active systemically and does not demonstrate organ-tropic effects or is capable of disrupting the physiology of more than one organ system. For example, autoimmunity may affect coagulation, the central nervous system, the cardiovascular system, and the musculoskeletal system (**Figure 2**). We do not have sufficient evidence yet on how individual biological mechanisms interact or whether one mechanism may propagate or spur the development of other mechanisms. It is plausible, for example, that anti-interferon antibodies may result in ineffective viral clearance and viral persistence, which may result in dysregulated immunity and autoantibody production, which may, in turn, result in neutrophil extracellular trap persistence and micro clotting. Each of these mechanisms may give rise to clinical sequelae and diverse phenotypes. The specific epidemiological and biological risk factors for the development of the putative biological mechanisms have not been fully elucidated but likely act through a complex interplay of genetic, host, pathogen, and acute infection factors with temporal evolution. There is a high likelihood that Long COVID represents a constellation of diverse clinical phenotypes resulting from complex pathways described by the interaction between multiple risk factors driving the development of single or multiple biological mechanisms capable of generating a diversity of clinical sequelae.

### Value of the proposed visualization

As scientific, clinical, and public health efforts for Long COVID escalate, an increasing level of interdisciplinary, inter-mural, and international coordination will be necessary. Highly defined, focused, and niche studies are helpful but they need to advance the field as a whole. The complexity of Long COVID demands systems biology as well as machine learning approaches and a constant awareness of the entire network of pathways implicated in the genesis and evolution of Long COVID. The visualization is a valuable reference tool to 1) locate and contextualize research efforts, 2) identify potential confounding mechanisms and pathways, 3) direct efforts for collaboration, and 4) provide a high-level perspective for individual scientific efforts. Research in this field will require well-characterized cohorts with longitudinal sampling and follow-up. Such cohorts represent global, and arguably communal, resources for understanding the fundamental building blocks of Long COVID and should be evaluated with due regard to the complexity of the condition, the vast network of pathways which may be implicated, and the dynamic nature of the interactions. The historical dependence on the follow-up of hospitalized, acute severe COVID-19 cohorts may not produce broadly generalizable information on Long COVID. The visualization is a call to step away from the simplified approach of siloed, highly defined research questions, toward sustained and collaborative efforts for a systems medicine and systems biology approach characterized by depth, breadth, and tight integration of the research components. Strengths of the associations in the visualization can be updated and monitored using natural language reading to broadly evaluate literature support or lack thereof for different connections and interactions, and with the use of machine learning algorithms, to distill the model into a more specific and actionable tool.

**Figure 3:**
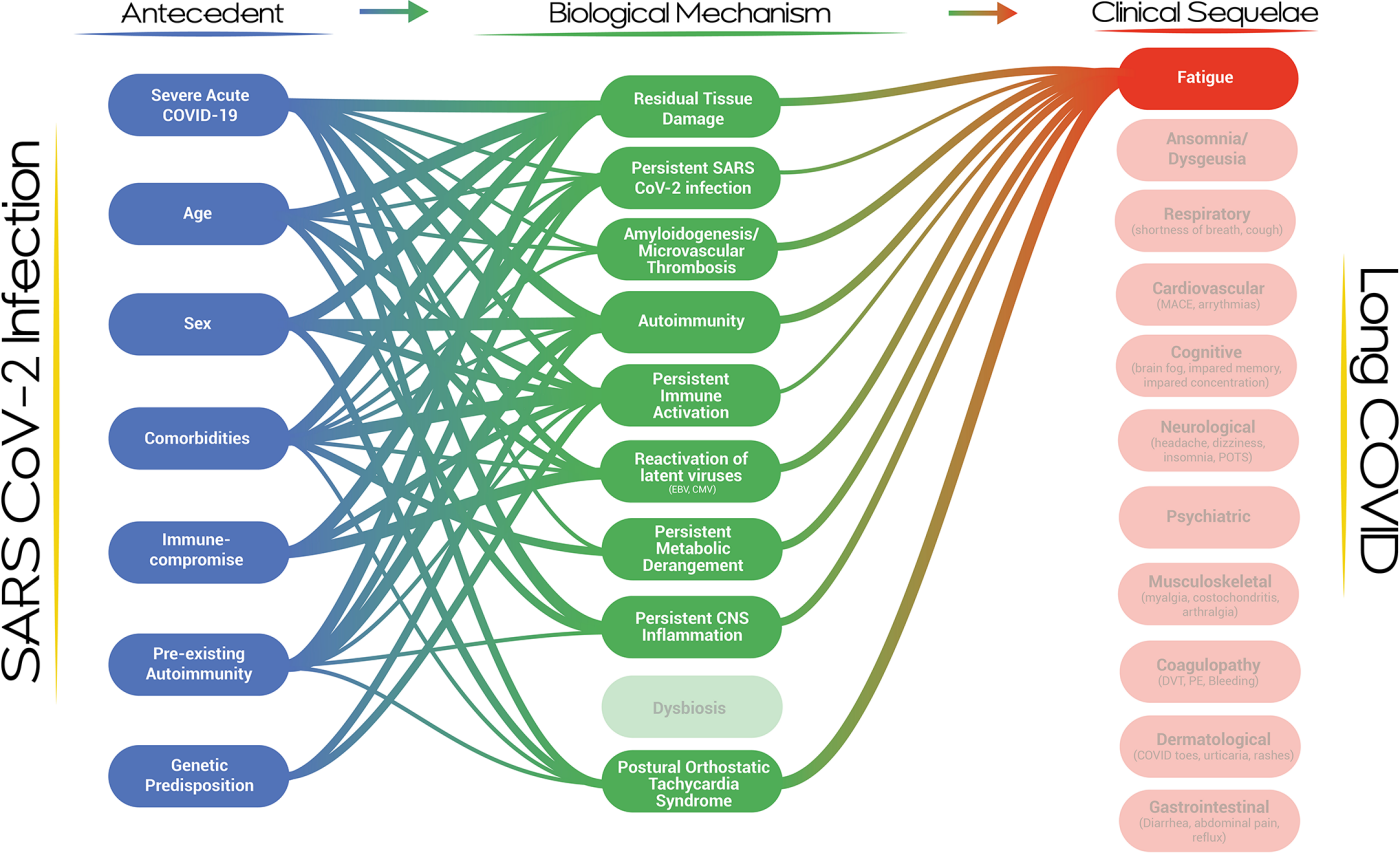
A network map of the risk factors and biological mechanisms associated with the development of fatigue in the context of Long COVID.

Furthermore, the visualization represents the entire cascade of events from acute infection to clinical phenotypes, including the phenotypic evolution and the risk for discrete clinical events. A clinical service for patients with post-viral fatigue would benefit from recognizing the biological mechanisms which could have led to the development of this phenotype and the risk factors that could contribute to the development of the biological mechanism/s in individual patients (**Figure 3**). Similarly, a scientist focused on understanding the role of autoimmunity should be aware of the competing explanatory biological mechanisms in the development of Long COVID and should be mindful of the Long COVID clinical and laboratory-based phenotypes which are under study (and equally so, those which are not). The visualization should guide existing and future opportunities for scientific and clinical development at each node and for the system overall. As a dynamic and iterative dashboard, the visualization should evolve alongside the evidence, incorporating new nodes and pathways as they become illuminated, and be developed into a framework for Long COVID clinical care and research. Lastly, the visualization is a reminder that the diverse clinical phenotypes of Long COVID, emanating from pathways of near-irreducible complexity, will require a holistic, multi-disciplinary, and comprehensive clinical and public health approach, that should further incorporate socioeconomic factors in the near future.

Future iterations of this visualization would benefit from incorporating kinetic aspects of each element, the impact of variants on driving particular pathways, the impact of vaccines and therapeutics interventions for acute COVID-19, and ultimately potential therapeutic interventions directed at specific biological mechanisms or clinical phenotypes.

## Conclusion

This perspective review provides a roadmap for the comprehensive characterization of LC cohorts, thereby creating a fundamental basis that can facilitate future research and clinical endeavors, potentially eliminating the threat of LC. The proposed visualization has the potential to contribute to the scientific community since the data can be updated as new knowledge emerges. Perhaps more importantly, the visualization may provide an innovative interactive dashboard for the state of LC research and may offer an alternative avenue to define, describe and address gaps in LC research on an international scale.

## Author contributions

RP, JN, KN, LS, and DW conceptualized the review. RP and LS performed the literature search and produced the first draft of the manuscript. All authors contributed to the article and approved the submitted version.

## References

[1] Zollner A Koch R Jukic A Pfister A Meyer M Rössler A. Postacute COVID-19 is characterized by gut viral antigen persistence in inflammatory bowel diseases. Gastroenterology (2022) 163(2):495–506. doi: 10.1053/j.gastro.2022.04.037 PMC9057012 35508284

[2] Chen C Haupert SR Zimmermann L Shi X Fritsche LG Mukherjee B. Global prevalence of post-coronavirus disease 2019 (COVID-19) condition or long COVID: a meta-analysis and systematic review. J Infect Dis (2022) 226(9):1593–607. doi: 10.1093/infdis/jiac136 PMC9047189 35429399

[3] Yoo SM Liu TC Motwani Y Sim MS Viswanathan N Samras N. Factors associated with post-acute sequelae of SARS-CoV-2 (PASC) after diagnosis of symptomatic COVID-19 in the inpatient and outpatient setting in a diverse cohort. J Gen Intern Med (2022) 2:1–8. doi: 10.1007/s11606-022-07523-3 PMC8989256 35391623

[4] Al-Aly Z Bowe B Xie Y. Long COVID after breakthrough SARS-CoV-2 infection. Nat Med (2022) 28(7):1461–7. doi: 10.1038/s41591-022-01840-0 PMC9307472 35614233

[5] Kenny G McCann K O’Brien C Savinelli S Tinago W Yousif O. Identification of distinct long COVID clinical phenotypes through cluster analysis of self-reported symptoms. Open Forum Infect Dis (2022) 9(4):ofac060. doi: 10.1093/ofid/ofac060 35265728 PMC8900926

[6] Berry C Mangion K. Multisystem involvement is common in post-COVID-19 syndrome. Nat Med (2022) 28(6):1139–40. doi: 10.1038/s41591-022-01838-8 PMC9125959 35606552

[7] Wise J. Covid-19: infection raises risk of diabetes and heart disease diagnoses in following weeks, study finds. BMJ (2022) 378:o1838. doi: 10.1136/BMJ.O1838 35868645

[8] Wang C Yu C Jing H Wu X Novakovic VA Xie R. Long COVID: the nature of thrombotic sequelae determines the necessity of early anticoagulation. Front Cell Infect Microbiol (2022) 12:861703/BIBTEX. doi: 10.3389/FCIMB.2022.861703/BIBTEX 35449732 PMC9016198

[9] Natarajan A Zlitni S Brooks EF Vance SE Dahlen A Hedlin H. Gastrointestinal symptoms and fecal shedding of SARS-CoV-2 RNA suggest prolonged gastrointestinal infection. Med (N Y) (2022) 3:371. doi: 10.1016/J.MEDJ.2022.04.001 PMC9005383 35434682

[10] Su Y Yuan D Chen DG Ng RH Wang K Choi J. Multiple early factors anticipate post-acute COVID-19 sequelae. Cell (2022) 185:881–95.e20. doi: 10.1016/J.CELL.2022.01.014 35216672 PMC8786632

[11] Pfaff ER Girvin AT Bennett TD Bhatia A Brooks IM Deer RR. Identifying who has long COVID in the USA: a machine learning approach using N3C data. Lancet Digit Health (2022) 4:e532. doi: 10.1016/S2589-7500(22)00048-6 35589549 PMC9110014

[12] Mehandru S Merad M. Pathological sequelae of long-haul COVID. Nat Immunol (2022) 23(2):194–202. doi: 10.1038/s41590-021-01104-y 35105985 PMC9127978

[13] Cutler DM Summers LH. The COVID-19 pandemic and the $16 trillion virus. JAMA (2020) 324:1495–6. doi: 10.1001/JAMA.2020.19759 PMC7604733 33044484

[14] Choutka J Jansari V Hornig M Iwasaki A. Unexplained post-acute infection syndromes. Nat Med (2022) 28(5):911–23. doi: 10.1038/s41591-022-01810-6 35585196

[15] Sudre CH Murray B Varsavsky T Graham MS Penfold RS Bowyer RC. Attributes and predictors of long COVID. Nat Med (2021) 27:626–31. doi: 10.1038/S41591-021-01292-Y PMC7611399 33692530

[16] Raveendran A. V.; misra, a. post COVID-19 syndrome (“Long COVID”) and diabetes: challenges in diagnosis and management. Diabetes Metab Syndr (2021) 15:102235. doi: 10.1016/J.DSX.2021.102235 34384972 PMC8317446

[17] Yomogida K Zhu S Rubino F Figueroa W Balanji N Holman E. Post-acute sequelae of SARS-CoV-2 infection among adults aged ≥18 years — long beach, California, April 1–December 10, 2020. MMWR Morb Mortal Wkly Rep (2021) 70:1274–7. doi: 10.15585/MMWR.MM7037A2 PMC8445372 34529639

[18] Desgranges F Tadini E Munting A Regina J Filippidis P Viala B. Post-COVID-19 syndrome in outpatients: a cohort study. J Gen Internal Med (2022) 2022:1–10. doi: 10.1007/S11606-021-07242-1 PMC8939498 35319081

[19] Perlis RH Green J Santillana M Lazer D Ognyanova K Simonson M. Persistence of symptoms up to 10 months following acute COVID-19 illness. medRxiv (2021) [Preprint]. doi: 10.1101/2021.03.07.21253072

[20] Augustin M Schommers P Stecher M Dewald F Gieselmann L Gruell H. Post-COVID syndrome in non-hospitalised patients with COVID-19: a longitudinal prospective cohort study. Lancet Regional Health - Europe (2021) 6:100122. doi: 10.1016/J.LANEPE.2021.100122 34027514 PMC8129613

[21] Naik S Haldar SN Soneja M Mundadan NG Garg P Mittal A. Post COVID-19 sequelae: a prospective observational study from northern India. Drug Discovery Ther (2021) 15:254–60. doi: 10.5582/DDT.2021.01093 34719599

[22] Matta J Wiernik E Robineau O Carrat F Touvier M Severi G. Association of self-reported COVID-19 infection and SARS-CoV-2 serology test results with persistent physical symptoms among French adults during the COVID-19 pandemic. JAMA Intern Med (2022) 182:19–25. doi: 10.1001/JAMAINTERNMED.2021.6454 34747982 PMC8576624

[23] Subramanian A Nirantharakumar K Hughes S Myles P Williams T Gokhale KM. Symptoms and risk factors for long COVID in non-hospitalized adults. Nat Med (2022) 28(8):1706–14. doi: 10.1038/s41591-022-01909-w PMC9388369 35879616

[24] Gold JE Okyay RA Licht WE Hurley DJ. Investigation of long COVID prevalence and its relationship to Epstein-Barr virus reactivation. Pathogens (2021) 10(6):763. doi: 10.3390/pathogens10060763 34204243 PMC8233978

[25] Ahmed S Jafri L Hoodbhoy Z Siddiqui I. Prognostic value of serum procalcitonin in COVID-19 patients: a systematic review. Indian J Crit Care Med (2021) 25:77. doi: 10.5005/JP-JOURNALS-10071-23706 33603306 PMC7874291

[26] Sigfrid L Drake TM Pauley E Jesudason EC Olliaro P Lim WS. Long covid in adults discharged from UK hospitals after covid-19: a prospective, multicentre cohort study using the ISARIC WHO clinical characterisation protocol. Lancet Regional Health - Europe (2021) 8:100186. doi: 10.1016/J.LANEPE.2021.100186/ATTACHMENT/63F64C27-94C1-482F-A63F-48FE8972B440/MMC12.DOCX 34386785 PMC8343377

[27] Mollica V Rizzo A Massari F. The pivotal role of TMPRSS2 in coronavirus disease 2019 and prostate cancer. Future Oncol (2020) 16:2029–33. doi: 10.2217/FON-2020-0571 PMC7359420 32658591

[28] Malkova A Kudlay D Kudryavtsev I Starshinova A Yablonskiy P Shoenfeld Y. Immunogenetic predictors of severe covid-19. mdpi.com (2021) 9:211. doi: 10.3390/vaccines9030211 PMC8001669 33802310

[29] Taneja V. Sex hormones determine immune response. Front Immunol (2018) 9:1931/FULL. doi: 10.3389/FIMMU.2018.01931/FULL 30210492 PMC6119719

[30] Takahashi T Ellingson MK Wong P Israelow B Lucas C Klein J. Sex differences in immune responses that underlie COVID-19 disease outcomes. Nature (2020) 588(7837):315–20. doi: 10.1038/s41586-020-2700-3 PMC7725931 32846427

[31] Lu Y Li X Geng D Mei N Wu PY Huang CC. Cerebral micro-structural changes in COVID-19 patients – an MRI-based 3-month follow-up study. EClinicalMedicine (2020) 25:100484. doi: 10.1016/J.ECLINM.2020.100484 32838240 PMC7396952

[32] van den Borst B Peters JB Brink M Schoon Y Bleeker-Rovers CP Schers H. Comprehensive health assessment 3 months after recovery from acute coronavirus disease 2019 (COVID-19). Clin Infect Dis (2021) 73:e1089. doi: 10.1093/CID/CIAA1750 33220049 PMC7717214

[33] Zhang X Wang F Shen Y Zhang X Cen Y Wang B. Symptoms and health outcomes among survivors of COVID-19 infection 1 year after discharge from hospitals in wuhan, China. JAMA Netw Open (2021) 4:e2127403–e2127403. doi: 10.1001/JAMANETWORKOPEN.2021.27403 34586367 PMC8482055

[34] Zeng F Dai C Cai P Wang J Xu L Li J. Comparison study of SARS-CoV-2 IgG antibody between Male and female COVID-19 patients: a possible reason underlying different outcome between sex. J Med Virol (2020) 92:2050–4. doi: 10.1002/JMV.25989 PMC7267228 32383183

[35] Beyerstedt S Casaro EB Rangel É.B. COVID-19: angiotensin-converting enzyme 2 (ACE2) expression and tissue susceptibility to SARS-CoV-2 infection. Eur J Clin Microbiol Infect Dis (2021) 40:905. doi: 10.1007/S10096-020-04138-6 33389262 PMC7778857

[36] Vrints CJM Krychtiuk KA van Craenenbroeck EM Segers VF Price S Heidbuchel H. Endothelialitis plays a central role in the pathophysiology of severe COVID-19 and its cardiovascular complications. Acta Cardiol (2020) 76:1. doi: 10.1080/00015385.2020.1846921 33208052 PMC7682384

[37] Voiriot G Oualha M Pierre A Salmon-Gandonnière C Gaudet A Jouan Y. Chronic critical illness and post-intensive care syndrome: from pathophysiology to clinical challenges. Ann Intensive Care (2022) 12(1):1–14. doi: 10.1186/S13613-022-01038-0 35779142 PMC9250584

[38] Vrettou CS Mantziou V Vassiliou AG Dimopoulou I Orfanos SE Kotanidou A. Post-intensive care syndrome in survivors from critical illness including COVID-19 patients: a narrative review. Life (Basel) (2022) 12(1):107. doi: 10.3390/life12010107 35054500 PMC8778667

[39] Klein SL Flanagan KL. Sex differences in immune responses. Nat Rev Immunol (2016) 16(10):626–38. doi: 10.1038/nri.2016.90 27546235

[40] Bajaj V Gadi N Spihlman AP Wu SC Choi CH Moulton VR. Aging, immunity, and COVID-19: how age influences the host immune response to coronavirus infections? Front Physiol (2021) 11:571416/BIBTEX. doi: 10.3389/FPHYS.2020.571416/BIBTEX 33510644 PMC7835928

[41] Spiering AE de Vries TJ. Why females do better: the X chromosomal TLR7 gene-dose effect in COVID-19. Front Immunol (2021) 12:756262/BIBTEX. doi: 10.3389/FIMMU.2021.756262/BIBTEX 34858409 PMC8632002

[42] Ciarambino T Para O Giordano M. Immune system and COVID-19 by sex differences and age. Women’s Health (2021) 17:17455065211022262. doi: 10.1177/17455065211022262 PMC8188967 34096383

[43] Al-Husinat L Nusir M Al-Gharaibeh H Alomari AA Smadi MM Battaglini D. Post-COVID-19 syndrome symptoms after mild and moderate SARS-CoV-2 infection. Front Med (Lausanne) (2022) 9:1017257/BIBTEX. doi: 10.3389/FMED.2022.1017257/BIBTEX 36262270 PMC9573938

[44] Maglietta G Diodati F Puntoni M Lazzarelli S Marcomini B Patrizi L. Prognostic factors for post-COVID-19 syndrome: a systematic review and meta-analysis. J Clin Med (2022) 11(6):1541. doi: 10.3390/jcm11061541 35329867 PMC8948827

[45] Thompson EJ Williams DM Walker AJ Mitchell RE Niedzwiedz CL Yang TC. Long COVID burden and risk factors in 10 UK longitudinal studies and electronic health records. Nat Commun (2022) 13(1):3528. doi: 10.1038/s41467-022-30836-0 35764621 PMC9240035

[46] Daitch V Yelin D Awwad M Guaraldi G Milić J Mussini C. Characteristics of long COVID among older adults: a cross-sectional study. Int J Infect Dis (2022) 125:287–93. doi: 10.1016/j.ijid.2022.09.035 36191820

[47] Notarte KI de Oliveira MHS Peligro PJ Velasco JV Macaranas I Ver AT. Age, sex and previous comorbidities as risk factors not associated with SARS-CoV-2 infection for long COVID-19: a systematic review and meta-analysis. J Clin Med (2022) 11(24):7314. doi: 10.3390/JCM11247314 36555931 PMC9787827

[48] Norredam M Hayward S Deal A Agyemang C Hargreaves S. Understanding and addressing long-COVID among migrants and ethnic minorities in Europe. Lancet Regional Health - Europe (2022) 19. doi: 10.1016/j.lanepe.2022.100427 PMC9241826 35789882

[49] Zeberg H Pääbo S. The major genetic risk factor for severe COVID-19 is inherited from neanderthals. Nature (2020) 587(7835):610–2. doi: 10.1038/s41586-020-2818-3 32998156

[50] Zeberg H Pääbo SA. Genomic region associated with protection against severe COVID-19 is inherited from neandertals. Proc Natl Acad Sci U.S.A. (2021) 118:e2026309118. doi: 10.1073/PNAS.2026309118/SUPPL_FILE/PNAS.2026309118.SAPP.PDF 33593941 PMC7936282

[51] Choi UY Kang JS Hwang YS Kim YJ. Oligoadenylate synthase-like (OASL) proteins: dual functions and associations with diseases. Exp Mol Med (2015) 47(3):e144–4. doi: 10.1038/emm.2014.110 PMC4351405 25744296

[52] Spinetti T Hirzel C Fux M Walti LN Schober P Stueber F. Reduced monocytic human leukocyte antigen-DR expression indicates immunosuppression in critically ill COVID-19 patients. Anesth Analg (2020) 131:993. doi: 10.1213/ANE.0000000000005044 32925314 PMC7288784

[53] Iturrieta-Zuazo I Rita CG García-Soidán A de Malet Pintos-Fonseca A Alonso-Alarcón N Pariente-Rodríguez R. Possible role of HLA class-I genotype in SARS-CoV-2 infection and progression: a pilot study in a cohort of covid-19 Spanish patients. Clin Immunol (2020) 219:108572. doi: 10.1016/J.CLIM.2020.108572 32810602 PMC7428760

[54] Truong TT Ryutov A Pandey U Yee R Goldberg L Bhojwani D. Increased viral variants in children and young adults with impaired humoral immunity and persistent SARS-CoV-2 infection: A consecutive case series. EBioMedicine (2021) 67:103355. doi: 10.1016/j.ebiom.2021.103355 33915337 PMC8072072

[55] Altuntas Aydin O Kumbasar Karaosmanoglu H Kart Yasar K. HIV/SARS-CoV-2 coinfected patients in Istanbul, Turkey. J Med Virol (2020) 92:2288–90. doi: 10.1002/JMV.25955 32347975

[56] Devresse A de Greef J Yombi JC Belkhir L Goffin E Kanaan N. Immunosuppression and SARS-CoV-2 infection in kidney transplant recipients. Transplant Direct (2022) 8:E1292. doi: 10.1097/TXD.0000000000001292 35187216 PMC8843373

[57] Pujari S Gaikwad S Chitalikar A Dabhade D Joshi K Bele V. Long-coronavirus disease among people living with HIV in Western India: an observational study. Immun Inflammation Dis (2021) 9:1037. doi: 10.1002/IID3.467 PMC8239760 34078004

[58] Pretorius E Vlok M Venter C Bezuidenhout JA Laubscher GJ Steenkamp J. Persistent clotting protein pathology in long COVID/Post-acute sequelae of COVID-19 (PASC) is accompanied by increased levels of antiplasmin. Cardiovasc Diabetol (2021) 20(1):1–8. doi: 10.1186/S12933-021-01359-7 PMC8381139 34425843

[59] Peluso MJ Deveau T-M Munter SE Ryder D Buck A Beck-Engeser G. Chronic viral coinfections differentially affect the likelihood of developing long COVID. Journal of Clinical Investigation. (2023). 133(3):1–1. doi: 10.1172/JCI163669 PMC9888380 36454631

[60] Cervia C Zurbuchen Y Taeschler P Ballouz T Menges D Hasler S. Immunoglobulin signature predicts risk of post-acute COVID-19 syndrome. Nat Commun (2022) 13(1):1–2. doi: 10.1038/S41467-021-27797-1 PMC8789854 35078982

[61] Herman JD Atyeo C Zur Y Cook CE Patel NJ Vanni KM. Impact of cross-coronavirus immunity in post-acute sequelae of COVID-19. medRxiv [Preprint] (2022). doi: 10.1101/2022.09.25.22280335

[62] Philip KEJ Buttery S Williams P Vijayakumar B Tonkin J Cumella A. Impact of COVID-19 on people with asthma: a mixed methods analysis from a UK wide survey. BMJ Open Respir Res (2022) 9:e001056. doi: 10.1136/BMJRESP-2021-001056 PMC8762134 35027428

[63] Aminian A Bena J Pantalone KM Burguera B. Association of obesity with postacute sequelae of COVID-19. Diabetes Obes Metab (2021) 23:2183–8. doi: 10.1111/DOM.14454 PMC8239834 34060194

[64] Debskiid M Tsampasianid V Haney S Blakely K Weston S Ntatsaki E. Post-COVID-19 syndrome risk factors and further use of health services in East England. PloS Global Public Health (2022) 2:e0001188. doi: 10.1371/JOURNAL.PGPH.0001188 36962824 PMC10022108

[65] Wang S Quan L Chavarro JE Slopen N Kubzansky LD Karestan KC. Associations of depression, anxiety, worry, perceived stress, and loneliness prior to infection with risk of post–COVID-19 conditions. JAMA Psychiatry (2022) 79(11):1081–91. doi: 10.1001/jamapsychiatry.2022.2640 PMC9453634 36069885

[66] Fernández-de-las-Peñas C Torres-Macho J Velasco-Arribas M Plaza-Canteli S Arias-Navalón JA Hernández-Barrera V. Preexisting hypertension is associated with a greater number of long-term post-COVID symptoms and poor sleep quality: a case–control study. J Hum Hypertension (2022) 36(6):582–4. doi: 10.1038/s41371-022-00660-6 PMC8853057 35173268

[67] Swank Z Senussi Y Manickas-Hill Z Yu XG Li JZ Alter G. Persistent circulating severe acute respiratory syndrome coronavirus 2 spike is associated with post-acute coronavirus disease 2019 sequelae. Clin Infect Dis (2023) 76(3):e487–90. doi: 10.1093/cid/ciac722 PMC10169416 36052466

[68] Cheung CCL Goh D Lim X Tien TZ Lim JCT Lee JN. Residual SARS-CoV-2 viral antigens detected in GI and hepatic tissues from five recovered patients with COVID-19. Gut (2022) 71:226–9. doi: 10.1136/gutjnl-2021-324570 34083386

[69] Brodin P Casari G Townsend L O’Farrelly C Tancevski I Löffler-Ragg J. Studying severe long COVID to understand post-infectious disorders beyond COVID-19. Nat Med (2022) 28(5):879–82. doi: 10.1038/s41591-022-01766-7 35383311

[70] Jacobs JJL. Persistent SARS-2 infections contribute to long COVID-19. Med Hypotheses (2021) 149:110538. doi: 10.1016/J.MEHY.2021.110538 33621843 PMC7884250

[71] Galán M Vigón L Fuertes D Murciano-Antón MA Casado-Fernández G Domínguez-Mateos S. Persistent overactive cytotoxic immune response in a Spanish cohort of individuals with long-COVID: identification of diagnostic biomarkers. Front Immunol (2022) 13:848886/BIBTEX. doi: 10.3389/FIMMU.2022.848886/BIBTEX 35401523 PMC8990790

[72] Rojas M Rodríguez Y Acosta-Ampudia Y Monsalve DM Zhu C Li Q-Z. Autoimmunity is a hallmark of post-COVID syndrome. J Transl Med (2022) 20:129. doi: 10.1186/s12967-022-03328-4 35296346 PMC8924736

[73] Woodruff MC Ramonell RP Haddad NS Anam FA Rudolph ME Walker TA. Dysregulated naive b cells and de Novo autoreactivity in severe COVID-19. Nature (2022) 611(7934):139–47. doi: 10.1038/s41586-022-05273-0 PMC9630115 36044993

[74] Sacchi MC Tamiazzo S Stobbione P Agatea L de Gaspari P Stecca A. SARS-CoV-2 infection as a trigger of autoimmune response. Clin Transl Sci (2021) 14:898–907. doi: 10.1111/CTS.12953 33306235 PMC8212749

[75] Klein J Wood J Jaycox J Lu P Dhodapkar RM Gehlhausen JR. Distinguishing features of long COVID identified through immune profiling. medRxiv [Preprint] (2022). doi: 10.1101/2022.08.09.22278592 PMC10620090 37748514

[76] Pretorius E Page MJ Engelbrecht L Ellis GC Kell DB. Substantial fibrin amyloidogenesis in type 2 diabetes assessed using amyloid-selective fluorescent stains. Cardiovasc Diabetol (2017) 16:1–4. doi: 10.1186/S12933-017-0624-5 PMC5668975 29096623

[77] Pretorius E Venter C Jacobus G Mediclinic L Maritha S Kotze J. Prevalence of symptoms, comorbidities, brin amyloid microclots and platelet pathology in individuals with long COVID/ post-acute sequelae of COVID-19 (PASC). Cardiovasc Diabetol (2022) 21(1):148. doi: 10.21203/rs.3.rs-1205453/v2 35933347 PMC9356426

[78] Pretorius E Venter C Laubscher GJ Kotze MJ Moremi K Oladejo S. Combined triple treatment of fibrin amyloid microclots and platelet pathology in individuals with long COVID/ post-acute sequelae of COVID-19 (PASC) can resolve their persistent symptoms. Research Square (2021). Preprint. doi: 10.21203/RS.3.RS-1205453/V1

[79] Almutairi MM Sivandzade F Albekairi TH Alqahtani F Cucullo L. Neuroinflammation and its impact on the pathogenesis of COVID-19. Front Med (Lausanne) (2021) 8:745789. doi: 10.3389/FMED.2021.745789 34901061 PMC8652056

[80] Malkova A Kudryavtsev I Starshinova A Kudlay D Zinchenko Y Glushkova A. Post COVID-19 syndrome in patients with Asymptomatic/Mild form. Pathogens (2021) 10(11):1408. doi: 10.3390/pathogens10111408 34832564 PMC8620929

[81] Hampshire A Trender W Chamberlain SR Jolly AE Grant JE Patrick F. Cognitive deficits in people who have recovered from COVID-19. EClinicalMedicine (2021) 39:101044. doi: 10.1016/J.ECLINM.2021.101044/ATTACHMENT/0675FA72-7493-4A17-AA19-4AD85BEB1BF0/MMC2.DOCX 34316551 PMC8298139

[82] Yong SJ. Persistent brainstem dysfunction in long-COVID: a. ACS Chem Neurosci (2021) 12:573. doi: 10.1021/ACSCHEMNEURO.0C00793 33538586

[83] Lukiw WJ Pogue A Hill JM. SARS-CoV-2 infectivity and neurological targets in the brain. Cell Mol Neurobiol (2022) 42:217. doi: 10.1007/S10571-020-00947-7 32840758 PMC7445393

[84] Ekdahl KN Teramura Y Asif S Jonsson N Magnusson PU Nilsson B. Thromboinflammation in therapeutic medicine. Adv Exp Med Biol (2015) 865:3–17. doi: 10.1007/978-3-319-18603-0_1 26306440

[85] Proal AD VanElzakker MB. Long COVID or post-acute sequelae of COVID-19 (PASC): an overview of biological factors that may contribute to persistent symptoms. Front Microbiol (2021) 12:698169/BIBTEX. doi: 10.3389/FMICB.2021.698169/BIBTEX 34248921 PMC8260991

[86] Chen W Pan JY. Anatomical and pathological observation and analysis of SARS and COVID-19: microthrombosis is the main cause of death. Biol Proced Online (2021) 23:1–12. doi: 10.1186/S12575-021-00142-Y/METRICS 33472576 PMC7816139

[87] Varga Z Flammer A Steiger P Lancet MH-T. Endothelial cell infection and endotheliitis in COVID-19. Lancet (2020) 395(10234):1417–18. doi: 10.1016/S0140-6736(20)30937-5 PMC7172722 32325026

[88] Song W FitzGerald G. COVID-19, microangiopathy, hemostatic activation, and complement. Am Soc Clin Investig (2020) 130(8):3950–3. doi: 10.1172/JCI140183 PMC7410042 32459663

[89] Nishiga M Wang D Han Y L. D. COVID-19 and cardiovascular disease: from basic mechanisms to clinical perspectives. nature (2020) 17(9):543–58. doi: 10.1038/s41569-020-0413-9 PMC7370876 32690910

[90] Chung MK Zidar DA Bristow MR Cameron SJ Chan T Harding C. COVID-19 and cardiovascular disease: from bench to bedside. Am Heart Assoc (2021) 128:1214–36. doi: 10.1161/CIRCRESAHA.121.317997 PMC8048382 33856918

[91] Xie Y Xu E Bowe B Al-Aly Z. Long-term cardiovascular outcomes of COVID-19. Nat Med (2022) 28(3):583–90. doi: 10.1038/s41591-022-01689-3 PMC8938267 35132265

[92] Katwa LC Mendoza C Clements M. CVD and COVID-19: emerging roles of cardiac fibroblasts and myofibroblasts. Cells (2022) 11:1316. doi: 10.3390/CELLS11081316 35455995 PMC9031661

[93] Guler SA Ebner L Aubry-Beigelman C Bridevaux PO Brutsche M Clarenbach C. Pulmonary function and radiological features 4 months after COVID-19: first results from the national prospective observational Swiss COVID-19 lung study. Eur Respir J (2021) 57(4). doi: 10.1183/13993003.03690-2020 PMC8082329 33419891

[94] Rai DK Sharma P Kumar R. Post covid 19 pulmonary fibrosis. is it real threat? Indian J Tuberculosis (2021) 68:330–3. doi: 10.1016/J.IJTB.2020.11.003 PMC7654356 34099197

[95] Ali RMM Ghonimy MBI. Post-COVID-19 pneumonia lung fibrosis: a worrisome sequelae in surviving patients. Egyptian J Radiol Nucl Med (2021) 52:1–8. doi: 10.1186/S43055-021-00484-3/FIGURES/8

[96] Saadat S Noureddini M Mahjoubin-Tehran M Nazemi S Shojaie L Aschner M. Pivotal role of TGF-β/Smad signaling in cardiac fibrosis: non-coding RNAs as effectual players. Front Cardiovasc Med (2020) 7:588347. doi: 10.3389/FCVM.2020.588347 33569393 PMC7868343

[97] Zatterale F Longo M Naderi J Raciti GA Desiderio A Miele C. Chronic adipose tissue inflammation linking obesity to insulin resistance and type 2 diabetes. Front Physiol (2020) 10:1607/BIBTEX. doi: 10.3389/FPHYS.2019.01607/BIBTEX 32063863 PMC7000657

[98] Raman B Bluemke DA Lüscher TF Neubauer S. Long COVID: post-acute sequelae of COVID-19 with a cardiovascular focus. Eur Heart J (2022) 43:1157. doi: 10.1093/EURHEARTJ/EHAC031 35176758 PMC8903393

[99] Peluso MJ Deveau T-M Munter SE Ryder DM Buck AM Beck-Engeser G. Chronic viral coinfections differentially affect the likelihood of developing long COVID. J Clin Invest (2023) 133(3):1–11. doi: 10.1172/JCI163669 PMC9888380 36454631

[100] Rohrhofer J Graninger M Lettenmaier L Schweighardt J Gentile SA Koidl L. Association between Epstein-Barr-Virus reactivation and development of long-COVID fatigue. Allergy (2023) 78:297–9. doi: 10.1111/ALL.15471 PMC9538037 35950630

[101] Apostolou E Rizwan M Moustardas P Sjögren P Bertilson BC Bragée B. Saliva antibody-fingerprint of reactivated latent viruses after Mild/Asymptomatic COVID-19 is unique in patients with myalgic-Encephalomyelitis/Chronic fatigue syndrome. Front Immunol (2022) 13:949787/BIBTEX. doi: 10.3389/FIMMU.2022.949787/BIBTEX 36341457 PMC9630598

[102] Holmes E Wist J Masuda R Lodge S Nitschke P Kimhofer T. Incomplete systemic recovery and metabolic phenoreversion in post-Acute-Phase nonhospitalized COVID-19 patients: implications for assessment of post-acute COVID-19 syndrome. J Proteome Res (2021) 20:3315–29. doi: 10.1021/ACS.JPROTEOME.1C00224/ASSET/IMAGES/LARGE/PR1C00224_0007.JPEG 34009992

[103] Guarnieri JW Dybas JM Fazelinia H Kim MS Frere J Zhang Y. Targeted down regulation of core mitochondrial genes during SARS-CoV-2 infection. bioRxiv (2022). [Preprint]. doi: 10.1101/2022.02.19.481089

[104] Snider JM You JK Wang X Snider AJ Hallmark B Zec MM. Group IIA secreted phospholipase A2 is associated with the pathobiology leading to COVID-19 mortality. J Clin Invest (2021) 131(19). doi: 10.1172/JCI149236 PMC8483752 34428181

[105] Wood E Hall KH Tate W. Role of mitochondria, oxidative stress and the response to antioxidants in myalgic Encephalomyelitis/Chronic fatigue syndrome: a possible approach to SARS-CoV-2 “Long-haulers”? Chronic Dis Transl Med (2021) 7:14–26. doi: 10.1016/J.CDTM.2020.11.002 33251031 PMC7680046

[106] Ajaz S McPhail MJ Singh KK Mujib S Trovato FM Napoli S. Mitochondrial metabolic manipulation by SARS-CoV-2 in peripheral blood mononuclear cells of patients with COVID-19. Am J Physiol Cell Physiol (2021) 320:C57–65. doi: 10.1152/AJPCELL.00426.2020/ASSET/IMAGES/LARGE/AJ-ACEL200031F005.JPEG PMC7816428 33151090

[107] Allali I Bakri Y Amzazi S Ghazal H. Gut-lung axis in COVID-19. Interdiscip Perspect Infect Dis (2021) 2021:202. doi: 10.1155/2021/6655380 PMC7979298 33777139

[108] Giannos P Prokopidis K. Gut dysbiosis and long COVID-19: feeling gutted. J Med Virol (2022) 94:2917. doi: 10.1002/JMV.27684 35233795 PMC9088471

[109] Su Q Lau RI Liu Q Chan FKL Ng SC. Post-acute COVID-19 syndrome and gut dysbiosis linger beyond 1 year after SARS-CoV-2 clearance. Gut (2022). doi: 10.1136/GUTJNL-2022-328319 35940857

[110] Haran JP Bradley E Zeamer AL Cincotta L Salive MC Dutta P. Inflammation-type dysbiosis of the oral microbiome associates with the duration of COVID-19 symptoms and long COVID. JCI Insight (2021) 6(20). doi: 10.1172/jci.insight.152346 PMC8564890 34403368

[111] Giron LB Peluso MJ Ding J Kenny G Zilberstein NF Koshy J. Markers of fungal translocation are elevated during post-acute sequelae of SARS-CoV-2 and induce NF-KB signaling. JCI Insight (2022) 7(15). doi: 10.1172/jci.insight.160989 PMC9462470 35727635

[112] Jackson CB Farzan M Chen B Choe H. Mechanisms of SARS-CoV-2 entry into cells. Nat Rev Mol Cell Biol (2021) 23(1):3–20. doi: 10.1038/s41580-021-00418-x 34611326 PMC8491763

[113] Schweighauser M Arseni D Bacioglu M Huang M Lövestam S Shi Y. Age-dependent formation of TMEM106B amyloid filaments in human brains. Nature (2022) 605:310–4. doi: 10.1038/S41586-022-04650-Z PMC9095482 35344985

[114] Nyström S Hammarström P. Amyloidogenesis of SARS-CoV-2 spike protein. bioRxiv (2021) 2021:472920. doi: 10.1101/2021.12.16.472920 PMC9136918 35579205

[115] Kavi L. Postural tachycardia syndrome and long COVID: an update. Br J Gen Pract (2022) 72:8–9. doi: 10.3399/BJGP22X718037 34972793 PMC8714530

[116] Chadda KR Blakey EE Huang CLH Jeevaratnam K. Long COVID-19 and postural orthostatic tachycardia SyndromeIs dysautonomia to be blamed? Front Cardiovasc Med (2022) 9:860198/XML/NLM. doi: 10.3389/FCVM.2022.860198/XML/NLM 35355961 PMC8959615

[117] Dani M Dirksen A Taraborrelli P Torocastro M Panagopoulos D Sutton R. Autonomic dysfunction in ‘Long COVID’: rationale, physiology and management strategies. Clin Med (2021) 21:e63. doi: 10.7861/CLINMED.2020-0896 PMC7850225 33243837

[118] World Health Organization. A clinical case definition of post COVID-19 condition by a Delphi consensus, 6 October 2021. Available at: https://www.who.int/publications/i/item/WHO-2019-nCoV-Post_COVID-19_condition-Clinical_case_definition-2021.1 (Accessed 3 June 2022).

[119] Bowe B Xie Y Al-Aly Z. Acute and postacute sequelae associated with SARS-CoV-2 reinfection. Nature medicine (2022) 10:1–8. doi: 10.1038/s41591-022-02051-3 PMC9671810 36357676

[120] Ballering A van Zon SKR olde Hartman TC Rosmalen JGM. Persistence of somatic symptoms after COVID-19 in the Netherlands: an observational cohort study. Lancet (2022) 400:452–61. doi: 10.1016/S0140-6736(22)01214-4 PMC9352274 35934007

[121] Evans RA Leavy OC Richardson M Elneima O McAuley HJC Shikotra A. Clinical characteristics with inflammation profiling of long COVID and association with 1-year recovery following hospitalisation in the UK: a prospective observational study. Lancet Respir Med (2022) 10:761–75. doi: 10.1016/S2213-2600(22)00127-8 PMC9034855 35472304

[122] Bungenberg J Humkamp K Hohenfeld C Rust MI Ermis U Dreher M. Long COVID-19: objectifying most self-reported neurological symptoms. Ann Clin Transl Neurol (2022) 9:141–54. doi: 10.1002/ACN3.51496 PMC8862437 35060361

[123] Mackay A. A paradigm for post-Covid-19 fatigue syndrome analogous to ME/CFS. Front Neurol (2021) 12:701419/BIBTEX. doi: 10.3389/FNEUR.2021.701419/BIBTEX 34408721 PMC8365156

[124] Calabria M García-Sánchez C Grunden N Pons C Arroyo JA Gómez-Anson B. Post-COVID-19 fatigue: the contribution of cognitive and neuropsychiatric symptoms. J Neurol (2022) 269:3990–9. doi: 10.1007/S00415-022-11141-8 PMC9055007 35488918

[125] Huang L Yao Q Gu X Wang Q Ren L Wang Y. 1-year outcomes in hospital survivors with COVID-19: a longitudinal cohort study. Lancet (2021) 398:747–58. doi: 10.1016/S0140-6736(21)01755-4/ATTACHMENT/5440AE6D-9DED-4100-8EE6-77F72BA95154/MMC1.PDF PMC8389999 34454673

[126] Myall KJ Mukherjee B Castanheira AM Lam JL Benedetti G Mak SM. Persistent post-COVID-19 interstitial lung disease. an observational study of corticosteroid treatment. Ann Am Thorac Soc (2021) 18:799–806. doi: 10.1513/ANNALSATS.202008-1002OC 33433263 PMC8086530

[127] Culebras M Loor K Sansano I Persiva Ó. Clofent D Polverino E. Histological findings in transbronchial cryobiopsies obtained from patients after COVID-19. Chest (2022) 161:647–50. doi: 10.1016/J.CHEST.2021.09.016 PMC8464080 34582842

[128] Matheson AM McIntosh MJ Kooner HK Lee J Desaigoudar V Bier E. Persistent 129 xe MRI pulmonary and CT vascular abnormalities in symptomatic individuals with post-acute COVID-19 syndrome. Radiology (2022) 305(2):466–76. doi: 10.1148/radiol.220492 PMC9272782 35762891

[129] Davis HE Assaf GS McCorkell L Wei H Low RJ Re’em Y. Characterizing long COVID in an international cohort: 7 months of symptoms and their impact. EClinicalMedicine (2021) 38:101019. doi: 10.1016/j.eclinm.2021.101019 34308300 PMC8280690

[130] Dryden M Mudara C Vika C Blumberg L Mayet N Cohen C. Post-COVID-19 condition 3 months after hospitalisation with SARS-CoV-2 in south Africa: a prospective cohort study. Lancet Glob Health (2022) 10:e1247–56. doi: 10.1016/S2214-109X(22)00286-8 PMC9363040 35961348

[131] Aranyó J Bazan V Lladós G Dominguez MJ Bisbal F Massanella M. Inappropriate sinus tachycardia in post-Covid-19 syndrome. Scientific Reports (2022) 12(1):298. doi: 10.1038/s41598-021-03831-6 34996973 PMC8741896

[132] Ståhlberg M Reistam U Fedorowski A Villacorta H Horiuchi Y Bax J. Post-COVID-19 tachycardia syndrome: a distinct phenotype of post-acute COVID-19 syndrome. Am J Med (2021) 134:1451–6. doi: 10.1016/j.amjmed.2021.07.004 PMC8356730 34390682

[133] Roca-Fernandez A Wamil M Telford A Carapella V Thomaides-Brears H Dennis A. Cardiovascular impairment in long COVID one year post-SARS-COV-2 infection. J Am Coll Cardiol (2022) 79:1312. doi: 10.1016/S0735-1097(22)02303-8

[134] Shi H Zuo Y Navaz S Harbaugh A Hoy CK Gandhi AA. Endothelial cell-activating antibodies in COVID-19. medRxiv (2022) 74(7):1132–8. doi: 10.1101/2021.01.18.21250041 PMC9082472 35174669

[135] Stein SR Ramelli SC Grazioli A Chung JY Singh M Yinda CK. SARS-CoV-2 infection and persistence in the human body and brain at autopsy. Nature (2022) 612(7941):758–63. doi: 10.1038/s41586-022-05542-y PMC9749650 36517603

[136] Camazón NV Teis A Membrive MJM Llibre C Bayés-Genís A Mateu L. Long COVID-19 and microvascular disease-related angina. Rev Esp Cardiol (Engl Ed) (2022) 75:444. doi: 10.1016/J.REC.2021.10.010 34824040 PMC8552551

[137] Mohamed MO Banerjee A. Long COVID and cardiovascular disease: a learning health system approach. Nat Rev Cardiol (2022) 19(5):287–8. doi: 10.1038/s41569-022-00697-7 PMC8943785 35332308

[138] Zuo Y Yalavarthi S Navaz S Hoy C Harbaugh A Gockman K. Autoantibodies stabilize neutrophil extracellular traps in COVID-19. JCI Insight (2021) 6(15):e150111. doi: 10.1172/jci.insight.150111 34166229 PMC8410057

[139] Katsoularis I Fonseca-Rodríguez O Farrington P Jerndal H Lundevaller EH Sund M. Risks of deep vein thrombosis, pulmonary embolism, and bleeding after covid-19: nationwide self-controlled cases series and matched cohort study. BMJ (2022) 377:e069590. doi: 10.1136/BMJ-2021-069590 35387772 PMC8984137

[140] Knight R Walker V Ip S Cooper JA Bolton T Keene S. Association of COVID-19 with major arterial and venous thrombotic diseases: a population-wide cohort study of 48 million adults in England and Wales. Circulation (2022) 146:892–906. doi: 10.1161/CIRCULATIONAHA.122.060785 36121907 PMC9484653

[141] Fernández-Castañeda A Lu P Geraghty AC Song E Lee M-H Wood J. Mild respiratory COVID can cause multi-lineage neural cell and myelin dysregulation. Cell (2022) 185:2452–68.e16. doi: 10.1016/J.CELL.2022.06.008 35768006 PMC9189143

[142] Douaud G Lee S Alfaro-Almagro F Arthofer C Wang C McCarthy P. SARS-CoV-2 is associated with changes in brain structure in UK biobank. Nature (2022) 604(7907):697–707. doi: 10.1038/s41586-022-04569-5 35255491 PMC9046077

[143] Hampshire A Chatfield DA MPhil AM Jolly A Trender W Hellyer PJ. Multivariate profile and acute-phase correlates of cognitive deficits in a COVID-19 hospitalised cohort. EClinicalMedicine (2022) 47:101417. doi: 10.1016/j.eclinm.2022.101417 35505938 PMC9048584

[144] Song E Bartley CM Chow RD Ngo TT Jiang R Zamecnik CR. Divergent and self-reactive immune responses in the CNS of COVID-19 patients with neurological symptoms. Cell Rep Med (2021) 2(5):100288. doi: 10.1016/j.xcrm.2021.100288 33969321 PMC8091032

[145] Bartley CM Johns C Ngo TT Dandekar R Loudermilk RL Alvarenga BD. Anti-SARS-CoV-2 and autoantibody profiles in the cerebrospinal fluid of 3 teenaged patients with COVID-19 and subacute neuropsychiatric symptoms. JAMA Neurol (2021) 78:1503–9. doi: 10.1001/JAMANEUROL.2021.3821 PMC8546622 34694339

[146] Merikanto I Dauvilliers Y Chung F Wing YK de Gennaro L Holzinger B. Sleep symptoms are essential features of long-COVID - comparing healthy controls with COVID-19 cases of different severity in the international COVID sleep study (ICOSS-II). J Sleep Res (2022) 32(1):e13754. doi: 10.1111/jsr.13754 36208038 PMC9874584

[147] Badenoch JB Rengasamy ER Watson C Jansen K Chakraborty S Sundaram RD. Persistent neuropsychiatric symptoms after COVID-19: a systematic review and meta-analysis. Brain Commun (2022) 4(1):fcab297. doi: 10.1093/braincomms/fcab297 35169700 PMC8833580

[148] Xu E Xie Y Al-Aly Z. Long-term neurological sequelae of SARS-CoV-2 infection. Nat Med (2022) 2022:1–2. doi: 10.1038/s41591-022-02018-4 PMC9528864 36192556

[149] Ignatova D Krasteva K Akabalieva K Alexiev S. Post-COVID-19 psychosis: cotard’s syndrome and potentially high risk of harm and self-harm in a first-onset acute and transient psychotic disorder after resolution of COVID-19 pneumonia. Early Interv Psychiatry (2022) 16(10):1159–62. doi: 10.1111/eip.13254 PMC8653068 34796667

[150] Gasnier M Choucha W Radiguer F Faulet T Chappell K Bougarel A. Comorbidity of long COVID and psychiatric disorders after a hospitalisation for COVID-19: a cross-sectional study. J Neurol Neurosurg Psychiatry (2022) 0:1–8. doi: 10.1136/JNNP-2021-328516 35953265

[151] Meringer H Mehandru S. Gastrointestinal post-acute COVID-19 syndrome. Nat Rev Gastroenterol Hepatol (2022) 19(6):345–6. doi: 10.1038/s41575-022-00611-z PMC8981882 35383321

[152] Rafiqul Islam SM Foysal MJ Hoque MN Mehedi HMH Rob MA Salauddin A. Dysbiosis of oral and gut microbiomes in SARS-CoV-2 infected patients in Bangladesh: elucidating the role of opportunistic gut microbes. Front Med (Lausanne) (2022) 9:821777/FULL. doi: 10.3389/FMED.2022.821777/FULL 35237631 PMC8882723

[153] Stokel-Walker C. How long does SARS-CoV-2 stay in the body? BMJ (2022) 377:o1555. doi: 10.1136/BMJ.O1555 35764342

[154] Karaarslan F Güneri FD Kardeş S. Long COVID: Rheumatologic/Musculoskeletal symptoms in hospitalized COVID-19 survivors at 3 and 6 months. Clin Rheumatol (2022) 41:289–96. doi: 10.1007/S10067-021-05942-X PMC8553494 34713356

[155] Tan C Lim R Yeow M Fong J Balakrishnan T. Tietze’s syndrome post-COVID-19 infection in an adult patient. Cureus (2022) 14(7). doi: 10.7759/cureus.27499 PMC10564091 37817896

[156] Collins RA Ray N Ratheal K Colon A. Severe post-COVID-19 costochondritis in children. Proc (Bayl Univ Med Cent) (2022) 35:56. doi: 10.1080/08998280.2021.1973274 34966216 PMC8477585

[157] Sapkota HR Nune A. Long COVID from rheumatology perspective — a narrative review. Clin Rheumatol (2022) 41:337. doi: 10.1007/S10067-021-06001-1 34845562 PMC8629735

[158] McMahon DE Gallman AE Hruza GJ Rosenbach M Lipoff JB Desai SR. Long COVID in the skin: a registry analysis of COVID-19 dermatological duration. Lancet Infect Dis (2021) 21:313. doi: 10.1016/S1473-3099(20)30986-5 33460566 PMC7836995

[159] André R Hsieh A Trellu LT. Chronic acral lesions (“COVID toes”): to add to long post- COVID-19 syndrome? Angiology (2022) 73:788–9. doi: 10.1177/00033197211068938/ASSET/IMAGES/LARGE/10.1177_00033197211068938-FIG1.JPEG PMC9411688 34979814

[160] Ayoubkhani D Khunti K Nafilyan V Maddox T Humberstone B Diamond I. Post-covid syndrome in individuals admitted to hospital with covid-19: retrospective cohort study. BMJ (2021) 372:n693. doi: 10.1136/BMJ.N693 33789877 PMC8010267

[161] Dennis A Cuthbertson DJ Wootton D Crooks M Gabbay M Eichert N. Multi-organ impairment and long COVID: a 1-year prospective, longitudinal cohort study. medRxiv (2022) 2022:3. doi: 10.1101/2022.03.18.22272607 PMC10041626 36787802

[162] Xie Y Al-Aly Z. Risks and burdens of incident diabetes in long COVID: a cohort study. Lancet Diabetes Endocrinol (2022) 10:311–21. doi: 10.1016/S2213-8587(22)00044-4 PMC8937253 35325624

[163] Yende S Parikh CR. Long COVID and kidney disease. Nat Rev Nephrol (2021) 17(12):792–3. doi: 10.1038/s41581-021-00487-3 PMC8427150 34504319

[164] Fierro NA. COVID-19 and the liver: what do we know after six months of the pandemic? Ann Hepatol (2020) 19:590–1. doi: 10.1016/J.AOHEP.2020.09.001 PMC7500273 32956871

[165] Roth NC Kim A Vitkovski T Xia J Ramirez G Bernstein D. Post-COVID-19 cholangiopathy: a novel entity. Am J Gastroenterol (2021) 116:1077–82. doi: 10.14309/AJG.0000000000001154 33464757

[166] Antonelli M Penfold RS Merino J Sudre CH Molteni E Berry S. Risk factors and disease profile of post-vaccination SARS-CoV-2 infection in UK users of the COVID symptom study app: a prospective, community-based, nested, case-control study. Lancet Infect Dis (2022) 22:43–55. doi: 10.1016/S1473-3099(21)00460-6 34480857 PMC8409907

[167] Azzolini E Levi R Sarti R Pozzi C Mollura M Mantovani A. Association between BNT162b2 vaccination and long COVID after infections not requiring hospitalization in health care workers. JAMA (2022) 328:676–8. doi: 10.1001/JAMA.2022.11691 PMC9250078 35796131

[168] Gao P Liu J Liu M. Effect of COVID-19 vaccines on reducing the risk of long COVID in the real world: a systematic review and meta-analysis. Int J Environ Res Public Health (2022) 19(19):12422. doi: 10.3390/ijerph191912422 36231717 PMC9566528

[169] Ayoubkhani D Bermingham C Pouwels KB Glickman M Nafilyan V Zaccardi F. Trajectory of long covid symptoms after covid-19 vaccination: community based cohort study. BMJ (2022) 377:e069676. doi: 10.1136/bmj-2021-069676 35584816 PMC9115603

